# Exploring the ternary interactions in Cu–ZnO–ZrO_2_ catalysts for efficient CO_2_ hydrogenation to methanol

**DOI:** 10.1038/s41467-019-09072-6

**Published:** 2019-03-11

**Authors:** Yuhao Wang, Shyam Kattel, Wengui Gao, Kongzhai Li, Ping Liu, Jingguang G. Chen, Hua Wang

**Affiliations:** 1 State Key Laboratory of Complex Nonferrous Metal Resources Clean Utilization Engineering, Kunming University of Science and Technology, Kunming, 650093 China; 2 Faculty of Metallurgical and Energy Engineering, Kunming University of Science and Technology, Kunming, 650093 China; 3 Chemistry Division, Brookhaven National Laboratory, Upton, NY 11973 USA; 4 Department of Earth and Environmental Engineering, Columbia University, New York, NY 10027 USA; 5 Department of Chemical Engineering, Columbia University, New York, NY 10027 USA; 6 School of Pharmacy and Chemistry, Dali University, Dali, 671003 China

## Abstract

The synergistic interaction among different components in complex catalysts is one of the crucial factors in determining catalytic performance. Here we report the interactions among the three components in controlling the catalytic performance of Cu–ZnO–ZrO_2_ (CZZ) catalyst for CO_2_ hydrogenation to methanol. The in situ diffuse reflectance infrared Fourier transform spectroscopy (DRIFTS) measurements under the activity test pressure (3 MPa) reveal that the CO_2_ hydrogenation to methanol on the CZZ catalysts follows the formate pathway. Density functional theory (DFT) calculations agree with the in situ DRIFTS measurements, showing that the ZnO–ZrO_2_ interfaces are the active sites for CO_2_ adsorption and conversion, while the presence of metallic Cu is also necessary to facilitate H_2_ dissociation and to provide hydrogen resource. The combined experiment and DFT results reveal that tuning the interaction between ZnO and ZrO_2_ can be considered as another important factor for designing high performance catalysts for methanol generation from CO_2_.

## Abstract (web summary)

Despite great efforts, the reaction mechanism of CO_2_ hydrogenation to methanol and the nature of the active sites on Cu–ZnO–ZrO_2_ (CZZ) catalysts are still under debate. Herein, the authors report the interactions among the three components in controlling the catalytic performance of CZZ catalyst for CO_2_ hydrogenation to methanol.

## Introduction

Methanol is an important chemical and energy carrier. The catalytic synthesis of methanol from CO_2_ (CO_2_ + 3H_2_ $\rightarrow$ CH_3_OH + H_2_O) has attracted considerable attention, because it is not only a potential way to mitigate CO_2_ emission but also an alternative process for methanol synthesis in chemical industry^1–5^. Cu/ZnO-based catalysts (e.g., Cu–ZnO–Al_2_O_3_ and Cu–ZnO–ZrO_2_) are the most widely studied for this process due to the high activity, high product selectivity, and low cost^2,3,6,7^. As a promising support and promoter, ZrO_2_ shows weak hydrophilic character in comparison to Al_2_O_3_, potentially inhibiting the poisoning effect of water on the active sites during methanol synthesis^8–10^. The presence of ZrO_2_ could also enhance the copper dispersion as well as the surface basicity, which should strongly affect the CO_2_ adsorption and methanol selectivity^11,12^. As a result, the Cu–ZnO–ZrO_2_ (CZZ) system has gained an increasing interest for its outstanding activity^1,8,9,11–17^.

Despite great efforts, the reaction mechanism of CO_2_ hydrogenation to methanol as well as the nature of the active sites on CZZ catalysts are still under debate and are not comprehensively understood due to the complexity of the ternary system^2,3,7,16,18,19^. In order to simplify the issues, binary catalyst models (e.g., Cu/ZnO and Cu/ZrO_2_) have been widely used to discuss the reaction mechanism. A general conclusion is that methanol synthesis over Cu-based catalysts is a structure-sensitive reaction and the synergetic effect between Cu and oxides is responsible for the enhanced activity^3,5,10,20–27^. The Cu/ZnO synergy is proposed to create active sites for CO_2_ and H_2_ conversion via a combination of defective Cu nanoparticles with ZnO thin overlayer (including induced morphological changes of Cu)^22,24,28^, junction effect at the Cu–ZnO interface (including enhanced electron transfer and increased generation of oxygen defects in the interface)^3,5,29–31^, or formation of a specific Cu–Zn surface alloy (including migration of Zn atoms over the Cu surface and incorporation of Zn atoms into the Cu step-edge sites)^7,19,21^. The Cu/ZrO_2_ synergy is mainly attributed to the formation of Cu–ZrO_2_ interfacial sites, which may promote the adsorption of CO_2_^10,26^, enhance the dissociation of H_2_ and spillover of atomic hydrogen^32,33^, bind the key reaction intermediates (*CO_2_, *CO, *HCO, and *H_2_CO) for further conversion^27^, and increase the turnover frequency^26^.

The role of ZnO–ZrO_2_ interaction is an ongoing debate. Recently, it was reported that the binary ZnO–ZrO_2_ oxide in the solid solution state also shows activity for CO_2_ hydrogenation to methanol^34^. The Zn-doped ZrO_2_ without the presence of Cu could achieve high CO_2_ conversion (10%) and methanol selectivity (86%) at high pressure (5 MPa) and high temperature (593 K). In comparison, CZZ catalysts with the presence of Cu usually could obtain high methanol yield at moderate conditions (3 MPa and 493–513 K, see the comparison in Supplementary Table 3). However, little is known about the atomic-level interaction among the three components in the Cu–ZnO–ZrO_2_ complex system, in particular under in situ reaction conditions.

Here, we report a mechanistic investigation of the ternary interactions of Cu/ZnO/ZrO_2_ in CO_2_ adsorption and activation for methanol synthesis by comparing the three-dimensional ordered macroporous (3DOM) catalysts with different particle size of ZnO. The 3DOM catalyst shows very high activity (18.2% CO_2_ conversion and 80.2% methanol selectivity obtained at 493 K and 3.0 MPa). The results from the in situ DRIFTS measurements at an activity test pressure (3 MPa) and DFT calculations indicate that the synergy among Cu, ZnO, and ZrO_2_ is essential to promote the CO_2_ conversion and methanol selectivity. The presence of Cu is necessary to allow the formation of active *H at the Cu–ZnO or Cu–ZrO_2_ interface for the final formation of methanol, while the ZnO–ZrO_2_ interface strongly enhances the activation and transformation of CO_2_ by promoting the hydrogenation of carbonate intermediate to more reactive species (e.g., formate and methoxy).

## Results

### Structural characterization

3DOM catalysts with different particle sizes of ZnO were prepared. The full details for all samples are collected in the Supplementary Information (SI) including the (HR) TEM images, size distributions of ZnO and ZrO_2_, XRD patterns, and specific surface areas of different samples. The average particle size of ZnO in the four 3DOM samples changes from 15 to 36 nm, and the ZrO_2_ particles are much smaller (3–4 nm) for all the samples (Supplementary Fig. 1). The specific surface areas (Supplementary Table 1) for the 3DOM samples are in the range of 32−35 m^2^/g.

**Fig. 1:**
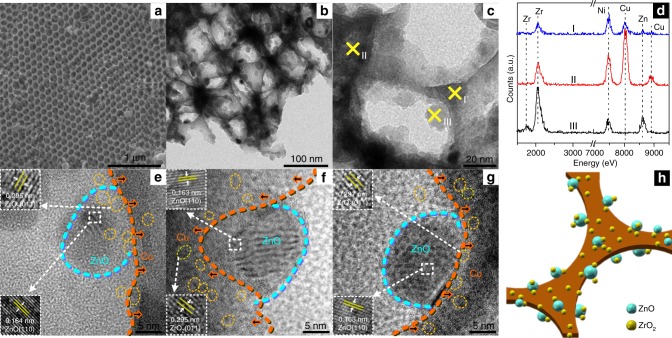
Morphological and compositive characterizations of a 3DOM CZZ catalyst (M-CZZ(16)). **a** SEM image, indicating an average pore size of 120 nm. **b**, **c** TEM images of the M-CZZ(16) sample, which shows the smallest ZnO particle size (15.8 nm) in the 3DOM catalyst series. **d** EDS of different points in **c**. **e**–**g** HRTEM images of M-CZZ(16). **h** Structural diagrammatic sketch of the macroporous catalysts

The EDS and TEM analyses (Fig. 1b–d) suggest that Cu makes up the 3DOM framework, and the ZnO particles are well dispersed on the wall of the macropores. The HRTEM images (Fig. 1e–g) show that t-ZrO_2_ nanoparticles (3.5 ± 1 nm, Supplementary Fig. 1), with a fringe spacing of 0.295 nm corresponding to the (011) plane, are highly dispersed on both the ZnO particles and Cu framework. The structural diagrammatic sketch of macroporous CZZ catalysts is shown in Fig. 1h. The XRD patterns, shown in Supplementary Fig. 2, reveal that the diffraction peak positions corresponding to Cu or ZrO_2_ are very similar for all the four samples. However, the differences in the peak widths of ZnO suggest changes of ZnO crystallite size in different samples, supporting the observation by TEM (see Supplementary Fig. 1).

### Catalytic performance and reaction intermediates

**Fig. 2:**
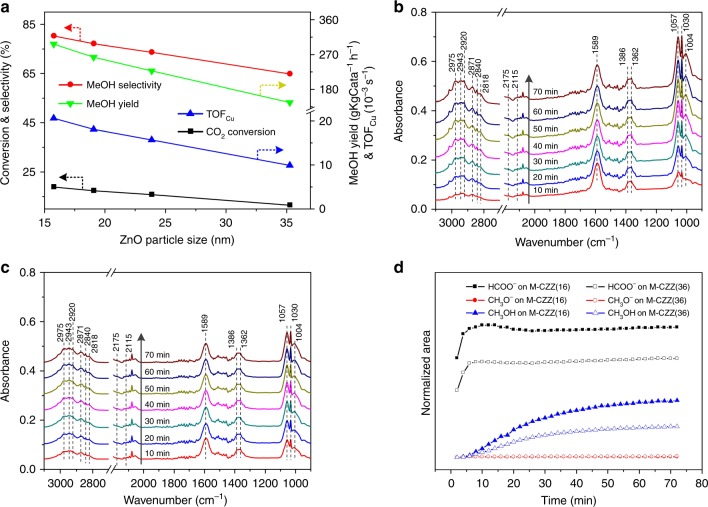
Catalytic performance and in situ DRIFT spectra of the CO_2_ + H_2_ reaction over different catalysts. **a** CO_2_ conversion, MeOH selectivity, MeOH yield and TOF values as a function of the ZnO particle sizes in different catalysts. **b** Evolution of the IR spectra over the M-CZZ(16) sample with time. **c** DRIFT spectra over the M-CZZ(36) sample. **d** Peak areas of generated intermediate species and methanol during the experiments: areas normalized to the values observed at the end of the transient. Reaction conditions for catalytic test: WHSV = 3 h^−1^, *T* = 493 K, CO_2_:H_2_ = 1:3, *P* = 3.0 MPa; Reaction conditions for TOF calculation: WHSV = 10 h^−1^, *T* = 493 K, CO_2_:H_2_ = 1:3, *P* = 3.0 MPa; Reaction conditions for in situ DRIFT spectra: gas flow rate = 40 mL/min, *T* = 493 K, CO_2_:H_2_ = 1:3, *P* = 3.0 MPa. Formate species (2870, 1589, 1386, and 1362 cm^−1^) and methanol (2975, 2943, 2920, 2871, 2840, and 2818 cm^−1^) can be observed over the two samples, especially for the M-CZZ(16) catalyst

Figure 2a shows the CO_2_ conversion, MeOH selectivity/yield and TOF value of methanol formation as a function of the ZnO particle size. The detailed data on the physicochemical and catalytic properties of different samples (e.g., diameter of Cu (*d*_Cu_), specific surface area of Cu (*S*_Cu_) and turnover frequency (TOF) values) are presented in Supplementary Tables 1, 2. As seen in Fig. 2a, the 3DOM catalyst with the smallest ZnO particles (M-CZZ(16) sample) possesses very high CO_2_ conversion (18.2%), methanol selectivity (80.2%) and methanol yield (297 g_MeOH_·Kg_Cata_^−1^ h^−1^). To our knowledge, this catalyst is the most active for CO_2_ hydrogenation to methanol among the CZZ catalysts under comparable conditions (Supplementary Table 3). It should be highlighted that the catalytic activity of the catalysts strongly relies on the ZnO particles. The CO_2_ conversion, methanol selectivity and the TOF value decrease with increasing ZnO particle size, suggesting that ZnO particles play a significant role in determining the catalytic performance.

Figure 2b–d show the DRIFT spectra obtained during CO_2_ hydrogenation at 493 K and 3 MPa over the M-CZZ(16) and M-CZZ(36) catalysts. For the M-CZZ(16) sample (Fig. 2b), strong bands at 1589, 1386, and 1362 cm^−1^ are observed, which are assigned to the *υ*_as_(OCO), *δ*(CH), and *υ*_s_(OCO) modes of formate species^27^, respectively. After the reaction proceeding for 10 min, vibrational bands at 1056, 1031, and 1005 cm^−1^ corresponding to the C–O stretch of methanol are observed, and bands at 2975, 2943, 2920, 2871, 2840, and 2818 cm^−1^, attributed to the C–H stretch of methanol, are also detected^35^, indicating the formation of methanol. The comparison of the in situ DRIFT spectra of methanol and methoxy is shown in the SI (see the Supplementary Fig. 4 and the related discussion). On the other hand, peaks at 2175 and 2115 cm^−1^, which are characteristic of gaseous CO, are also observed, indicating the occurrence of the reverse water-gas shift (RWGS) reaction. The weak and oscillating bands located in the range of 1400–1800 cm^−1^ are attributed to water vapor, which originates from the RWGS and methanol synthesis reactions^36^. In Fig. 2c, the corresponding bands observed over M-CZZ(16) are also detected over the M-CZZ(36) sample, but the band intensities of formate species and methanol are much weaker. This indicates that the particle size of ZnO affects the formation and evolution of intermediate species, and smaller ZnO particles enhance the formation of formate and methanol.

The temporal evolution of the principal surface species (formate, methoxy, and methanol) on both samples during the CO_2_ hydrogenation process are presented in Fig. 2d. The concentration of formate increases sharply at the beginning of the reaction, which is followed by a slight increase of methanol. This indicates that the formation of formate is very fast, while the generation of methanol needs an induction period. It should be highlighted that the methoxy species, which is a common intermediate observed by in situ IR under atmospheric or lower pressures^27,34^, is almost absent over the present samples. To identify the relationship between formate and methoxy species, the in situ DRIFTS experiment under atmospheric pressure is also performed, and the results are shown in Supplementary Fig. 5.

After the catalysts were pre-treated in flowing pure CO_2_ for 10 min at 493 K, transient response spectra were recorded with switching H_2_ into the reactor chamber. As shown in Supplementary Figs. 5a and b, before the switching, only peaks at ca. 1522 and 1352 cm^−1^ related to monodentate (m-CO_3_^2−^) and bidentate (b-CO_3_^2−^) carbonate species, as well as the peak at 1075 cm^−1^ assigned to carbonate ion (CO_3_^2−^), are detected^37,38^. The bands for formate species located at ca. 2972, 2878, 1588, 1384, and 1367 cm^−1^ appear as soon as H_2_ is introduced, while the peaks attributed to carbonate species almost disappear. This indicates that the carbonate could rapidly reacts with H to form formate.

It is also observed that the bands of formate slightly decrease in intensity with time, and the C–H (2930 and 2821 cm^−1^) and C–O (1145 and 1045 cm^−1^) stretching features^27^ attributed to the methoxy gradually increase in intensity. This suggests that methoxy should originate from the hydrogenation of formate species. After the reaction proceeds for 10 min, the bands related to water vapor (see the insert in Supplementary Figs. 5a and 5b) located in the range of 1400–1800 cm^−1^ are observed^36^, which is likely produced from the hydrogenation of formate. The evolution of surface species over catalysts during the onset of switching feed gas from CO_2_ to H_2_ after CO_2_ adsorption is presented in Supplementary Fig. 4c. It is clear that the intensity of methoxy slightly increases with the decrease in the formate intensity, evidencing the conversion of formate to methoxy. It is also noted that no CO intermediate is detected during the DRIFTS experiment under atmospheric pressure (see Supplementary Fig. 5), which is inconsistent with the formation of CO in the CO_2_ hydrogenation at 3.0 MPa (see Fig. 2). This reveals that the reaction pressure also affects the production of CO from CO_2_.

To eliminate the effect of 3DOM structure on the adsorption and catalytic activity, a series of conventional samples with various particle size of ZnO but comparable Cu and ZrO_2_ particle sizes were also prepared (which were labeled as nonporous samples, N-CZZ). All the characterization, catalytic tests and in situ DRIFT experiments, which were performed on the 3DOM samples, were also performed on the N-CZZ catalysts. The results are shown in Supplementary Figs. 6–9 and Supplementary Tables 1 and 2. It is found that the catalytic performance and the evolution of the intermediate species during the reaction also strongly rely on the particle size of ZnO. This suggests that the size effect of ZnO is a common phenomenon for the CZZ catalyst system.

### Comparison on Cu–ZnO, Cu–ZrO_2_, and ZnO–ZrO_2_ interactions

As observed above, the ZnO particle size strongly affects the CO_2_ adsorption, the formation of intermediate species and the methanol generation over the CZZ catalysts. On the other hand, the microstructures of Cu particles (e.g., Cu dispersion, Cu specific surface area and Cu particle size) in all the samples are similar (see Supplementary Table 1), and there is no direct correlation between the TOF values or methanol yields and the Cu surface area or Cu dispersion (see Supplementary Table 2). This suggests that the determining factor for the catalytic performance should be more complicated than the independent Cu related species. The size effect of ZnO may be related to the ZnO–ZrO_2_ interaction. To identify the roles of Cu–ZnO, Cu–ZrO_2_, and ZnO–ZrO_2_ interactions in CO_2_ adsorption and conversion, in situ DRIFT experiments were performed on all the three binary samples with switching the feed gas from CO_2_ to H_2_. The crystallite sizes of different phases in the three samples are controlled in a comparable range to ensure the comparability (see Supplementary Fig. 10).

**Fig. 3:**
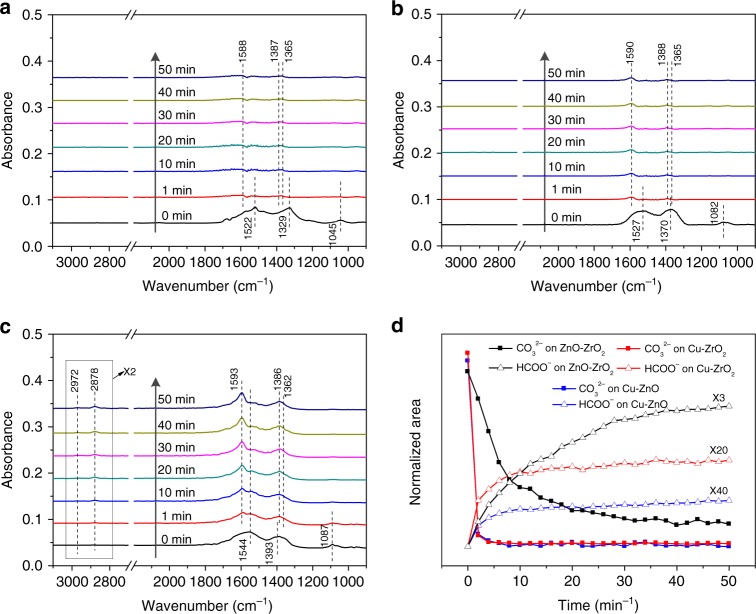
Comparison on the surface species on Cu–ZnO, Cu–ZrO_2_, and ZnO–ZrO_2_ systems in the designed conditions. **a**–**c** In situ DRIFT spectra over **a** Cu–ZnO, **b** Cu–ZrO_2_, and **c** ZnO–ZrO_2_ materials at 493 K after switching feed gas from CO_2_ (CO_2_ has been loaded into the camber for 10 min) to H_2_ with a flow rate of 40 mL/min under atmosphere pressure. **d** Peak areas of generated intermediate species during the experiments: areas normalized to the values observed at the end of the transient. Only the ZnO–ZrO_2_ sample shows obvious formation of formate species (2972, 2878, 1593, 1386, and 1362 cm^−1^) from carbonate species when the feed gas was switched from CO_2_ to H_2_

Figure 3a shows the transient evolution of the principal surface species over the Cu–ZnO sample. In the CO_2_ atmosphere, the carbonate species (1522, 1329, and 1045 cm^−1^) are observed. After switching gas to H_2_, the carbonate species disappear and very weak formate bands (1588, 1387, and 1365 cm^−1^) are detected. Similar phenomena are also observed on the Cu–ZrO_2_ sample (Fig. 3b). This indicates that the conversion of carbonate species to formate species is very difficult on these two binary samples. In contrast, abundant formate species (2972, 2878, 1593, 1386, and 1362 cm^−1^) are formed after switching CO_2_ to H_2_ on the ZnO–ZrO_2_ sample (Fig. 3c), concurrent with the decrease of carbonate species (Fig. 3d). The in situ DRIFT experiments at atmosphere or 3 MPa in the CO_2_/H_2_ mixture also reveal that only the ZnO–ZrO_2_ system shows relatively high intensity of formate bands among the three samples (Supplementary Figs. 11, 12). But it should be noted that no methanol is detected over the ZnO–ZrO_2_ sample at the activity test condition without the presence of Cu (see Supplementary Fig. 12c). These phenomena suggest that the ZnO–ZrO_2_ interface should be the active sites for CO_2_ adsorption to carbonate species and its subsequent conversion to formate and the Cu species may contribute to the further hydrogenation process.

To further study the effect of the ZnO–ZrO_2_ interaction on the CO_2_ adsorption and formate formation, a series of ZnO–ZrO_2_ samples with different particle sizes of ZnO and similar particle sizes (average sizes of 3–4 nm) of ZrO_2_ were prepared. The typical TEM images of different samples are shown in Supplementary Fig. 14. The average particle sizes for the three samples (ZnO(15)–ZrO_2_, ZnO (21)–ZrO_2_, and ZnO(42)–ZrO_2_) are 15.1, 21.4, and 42.2 nm, respectively.

**Fig. 4:**
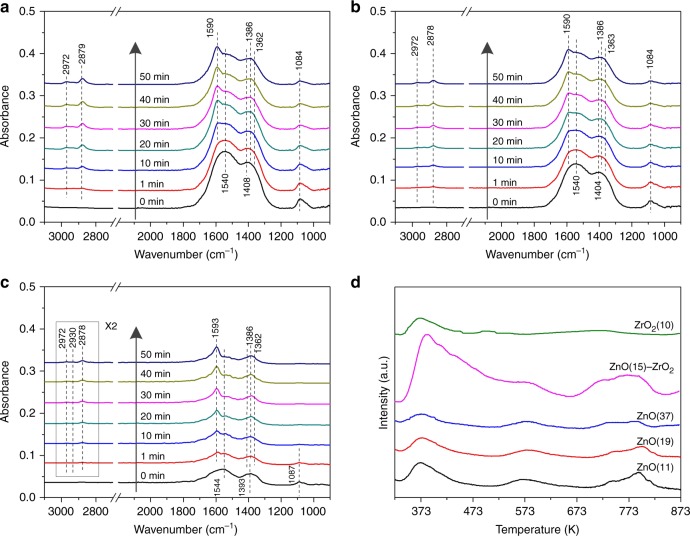
Characterization of the surface species on the ZnO–ZrO_2_ samples with different particles size of ZnO. In situ DRIFT spectra of **a** ZnO(15)–ZrO_2_, **b** ZnO(21)–ZrO_2_, and **c** ZnO(42)–ZrO_2_ at 493 K after switching the feed gas from CO_2_ to H_2_ with a flow rate of 40 mL/min under atmosphere pressure. **d** CO_2_-TPD patterns of ZnO(15)–ZrO_2_, pure ZnO with different particle sizes and pure ZrO_2_ with an average particle size of 10 nm

Figure 4a–c represent the in situ DRIFT spectra over the samples when switching the feed gas from CO_2_ to H_2_. As shown in Fig. 4a, only carbonate species (1540, 1408, and 1084 cm^−1^) are observed over the ZnO(15)–ZrO_2_ sample in CO_2_. After switching the feed gas from CO_2_ to H_2_, the formate bands (2972, 2878, 1590, 1386, and 1363 cm^−1^) appear immediately with the decrease of carbonate, indicating the hydrogenation of carbonate to formate. Figures 4b, c show similar trend over the ZnO(21)–ZrO_2_ and ZnO(42)–ZrO_2_ samples. It is notable that the formation of carbonate and its further conversion to formate is inversely proportion to the ZnO particle size. Smaller ZnO particles promote the adsorption of CO_2_ and its further conversion to formate, which may be attributed to the more abundance of the ZnO–ZrO_2_ interface.

In order to further understand the interaction between ZnO and ZrO_2_, we have performed CO_2_-TPD experiments over the ZnO(15)–ZrO_2_ sample, pure ZnO particles with different average sizes (11, 19, and 37 nm) and pure ZrO_2_ with an average particle size of 10 nm. As shown in Fig. 4d, the CO_2_ adsorption capacity slightly decreases with increasing ZnO particle size. In comparison, the ZnO(15)–ZrO_2_ sample shows much higher CO_2_ adsorption peak at low temperatures (373–473 K) than the pure ZrO_2_ or ZnO regardless of the particle size, indicating that the interaction between ZnO and ZrO_2_ strongly improves the CO_2_ adsorption capacity.

Oxygen vacancy also plays a very important role in the hydrogenation of CO or CO_2_ to methanol, which can adsorb and activate reactive gases and stabilize the reaction intermediates, elevating the catalytic activity^21,22,24^. Kurtz et al.^39^ found that the oxygen vacancy in the ZnO/Al_2_O_3_ catalyst is the active site for hydrogenation of CO to methanol. For Cu–ZnO catalysts in CO_2_ hydrogenation, it is proposed that the activity is correlated to the number of oxygen defects between Cu and ZnO particles^22^. In the case of Cu–ZrO_2_ catalysts, the oxygen vacancies created by the evolution of ZrO_2_ phase also strongly affect the methanol formation. According to the literatures^40,41^, catalysts with monoclinic ZrO_2_ (m-ZrO_2_) are nearly an order of magnitude more active for methanol synthesis from CO_2_ than catalysts with the same Cu surface density deposited on tetragonal ZrO_2_ (t-ZrO_2_) due to the relatively higher concentration of oxygen vacancies. The formation of oxygen vacancies resulting from the ZnO–ZrO_2_ interaction is evidenced by comparing the XPS spectra of ZnO/ZrO_2_ sample with that of pure ZnO or ZrO_2_ (see Supplementary Fig. 17 and the related discussions). Such oxygen vacancies can be the active sites for CO_2_ adsorption.

For CO_2_ hydrogenation to methanol over Cu-based catalysts, formate pathway (featured by formate as the main intermediate) and CO-Hydro pathway (featured by the CO intermediate produced via RWGS) are considered as two major reaction mechanisms^5^. The formate pathway is defined by the following two conditions: (1) formate can be quickly generated and not easily decomposed into CO and (2) formate should not be excessively stable and can be hydrogenated to methoxy. In the present work, it can be seen from Supplementary Figs. 5 and 9 that the carbonate is rapidly converted to formate in the presence of H_2_ and no CO is detected in this process. Meanwhile, the DRIFTS intensity of *CH_3_O increases along with the decrease of the *HCOO signal. It is also found that the samples with more detectable formate in the DRIFTS experiments show higher activity for methanol formation (see Fig. 2), indicating that the formate (instead of being a spectator) should be an important intermediate for the formation of methanol. By contrast, no direct correlation is found between the formation of CO and the yield of methanol. These phenomena suggest that the CO_2_ hydrogenation over the present CZZ catalysts follows the formate pathway.

### Density functional theory studies

DFT calculations were performed to understand the catalytic behavior of the ZrO_2_/ZnO interface during CO_2_ conversion to CH_3_OH. Herein, the ZrO_2_/ZnO interface is modeled by depositing a small ZrO_2_ cluster on the ZnO(11$\bar 2$0) surface (for details see computational method section in the SI). The surface O atoms in our model were saturated with H atoms to account for the possible H spillover from Cu to oxide nanoparticles due to facile H_2_ dissociation on Cu, as predicted by DFT calculations and observed in our experiments.

**Fig. 5:**
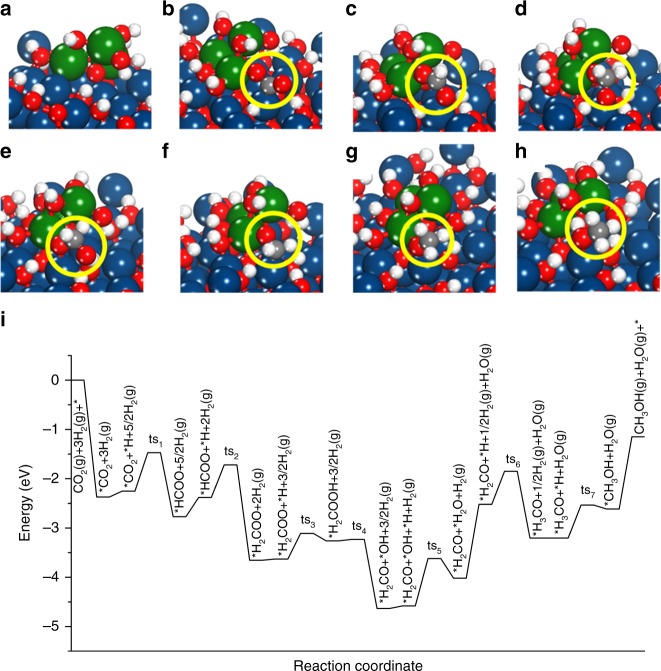
Density functional theory results. **a**–**h** DFT optimized configurations of **a** ZrO_2_/ZnO(11$\bar 2$0) and **b** *CO_2_, **c** *HCOO, **d** *H_2_COO, **e** *H_2_COOH, **f** *H_2_CO, **g** *H_3_CO, and **h** *CH_3_OH adsorbed at the ZrO_2_/ZnO(11$\bar 2$0) interface. *(X) indicates adsorbed species. The reaction intermediates are shown inside the yellow circle. Dark blue: Zn, green: Zr, red: O, gray: C and white: H. **i** Energy profile for CO_2_ hydrogenation to CH_3_OH at the ZrO_2_/ZnO(11$\bar 2$0) interface via the formate pathway. “ts” corresponds to the transition state

Figure 5a and Supplementary Fig. 25 show the reaction intermediates that are involved in CO_2_ hydrogenation, and the structure of the intermediates with different views can be found in Supplementary Fig. 26. The binding energy (−2.32 eV) for CO_2_ adsorption on ZrO_2_–ZnO(11$\bar 2$0) is much stronger than that for ZnO–Cu(111) (−0.13 eV), ZrO_2_–Cu(111) (−1.18 eV), ZrO_2_ cluster on ZnO(11$\bar 2$0) (−1.95 eV), and ZnO(11$\bar 2$0) (−1.94 eV), indicating that the activation and transformation of CO_2_ prefer to occur at the ZrO_2_/ZnO interface.

Previously, the role of ZnO for CO_2_ hydrogenation has been debated and previous studies have suggested that ZnO acts as an active phase for CO_2_/CO hydrogenation to CH_3_OH^42,43^. The CO_2_ $\rightarrow$ CH_3_OH conversion on ZnO(11$\bar 2$0) is further investigated using DFT. The results suggest that the initial hydrogenation of CO_2_ on ZnO(11$\bar 2$0) likely proceeds via the formation of the *HOCO intermediate since *HOCO formation (Δ*E* = −0.16 eV and *E*_a_ = 0.15 eV) is kinetically more favorable than *HCOO formation (Δ*E* = −1.42 eV and *E*_a_ = 0.32 eV). The *HOCO dissociation produces a key reaction intermediate, *CO, along the RWGS + CO-Hydro pathway for CH_3_OH synthesis^27,44^. However, in consideration of the entropic contribution, *CO may not be stable on the ZnO(11$\bar 2$0) surface under reaction conditions with a BE of −1.33 eV, preferring desorption rather than its further hydrogenation. Thus, CO is predicted to be the product of CO_2_ hydrogenation on ZnO(11$\bar 2$0), which agrees well with the corresponding experimental observation of CO but not CH_3_OH as a product of CO_2_ hydrogenation on ZnO(11$\bar 2$0) (see Supplementary Figs. 18, 19).

The DFT optimized geometries at the ZrO_2_/ZnO interface were then used to calculate the reaction energy (Δ*E*) and activation energy (*E*_a_) of each elementary step involved in CO_2_ hydrogenation to CH_3_OH via the formate pathway, which was identified as an active intermediate by in situ DRIFTS for the CZZ catalysts.

Figure 5b shows the energy profile for the formation of CH_3_OH from CO_2_ and H_2_ along the formate pathway. The initial step in CO_2_ hydrogenation, i.e., the formation of the *HCOO intermediate is exothermic with Δ*E* of −0.52 eV and *E*_a_ of 0.78 eV. Further hydrogenations of *HCOO leads to the formations of *H_2_COO (Δ*E* = −1.27 eV; *E*_a_ = 0.66 eV) and *H_2_COOH (Δ*E* = 0.37 eV; *E*_a_ = 0.52 eV). Compared with *HCOO hydrogenation to *H_2_COO, the *H assisted dissociation to HCO (*HCOO + *H → *HCO + *OH, Δ*E* = −0.44 eV; *E*_a_ = 1.45 eV) and subsequently to *CO (*HCO + * $\rightarrow$ *CO + *H, Δ*E* = 1.51 eV; *E*_a_ = 1.63 eV) is kinetically more difficult at the ZnO/ZrO_2_ interface. Thus, it suggests that the formation of *CO from *HCOO is highly unlikely, in agreement with the experimental results (Supplementary Figs. 5 and 9). The ZnO–ZrO_2_ interface facilities not only the activation of *HCOO, the rate-limiting step for CO_2_ hydrogenation at the ZnO–Cu interface^3^ via lowering the barrier from 0.85 to 0.66 eV, but also the formation of *H_2_COOH, a highly activated step via lowering the barrier from 0.90 to 0.52 $\rightarrow$eV. This agrees well with the in situ DRIFTS results (Supplementary Fig. 5), showing a fast conversion of *HCOO to *H_3_CO over the CZZ catalyst. *H_2_COOH is the precursor for C–O bond breaking as in the case of Cu/ZnO, however, the corresponding barrier (Δ*E* = −1.37 eV; *E*_a_ = 0.03 eV) is much lower on ZnO–ZrO_2_ than on Cu/ZnO (*E*_a_ = 0.49 eV)^3^. The hydrogenation of dissociated *H_2_CO results in the formation of *H_3_CO (Δ*E* = −0.68 eV; *E*_a_ = 0.67 eV) and eventually the production of *CH_3_OH (Δ*E* = 0.59 eV; *E*_a_ = 0.67 eV). Similar promotion is also observed when compared to Cu/ZrO_2_, which shows more difficult activations of *HCOO to *HCOOH (∆*E* = 0.70 eV, *E*_a_ = 0.80 eV) and *H_2_COOH dissociation to *H_2_CO and *OH (∆*E* = 0.67 eV, *E*_a_ = 1.32 eV) than ZnO–ZrO_2_^27^.

For the reaction pathway at the ZnO–ZrO_2_ interface, the dissociated *OH is removed from the surface by the formation of H_2_O, which is an endothermic process (Δ*E* = 0.56 eV) and has to overcome the highest barrier of 0.96 eV along the pathway. In contrast, it is a facile step at the ZnO–Cu interface (∆*E* = −0.43 eV; *E*_a_ = 0.22 eV)^3^. Therefore, the potential synergy between the interfaces of ZnO–ZrO_2_ and ZnO–Cu might also play an important role in promoting the removal of surface *OH to regenerate the active sites. According to the DFT calculated energy barriers, the formation of ZnO–ZrO_2_ interface is able to promote the *HCOO activations, which have been identified to control the reaction rates at the Cu/ZnO and Cu/ZrO_2_ interfaces during CO_2_ hydrogenation; yet the removal of *OH from the surface to produce gas phase H_2_O can be problematic at lower temperatures. Nevertheless, the barrier is <1 eV and is expected to be partially overcome at *T* = 493 K to maintain sufficient amount of active sites for hydrogenation of CO_2_ to CH_3_OH.

The Cu component was not specifically considered in the present DFT calculations. At the ZnO–ZrO_2_ interface, the H_2_ dissociative adsorption is an endothermic process (Δ*E* = 0.47 eV), which is less favorable than that at the -Cu/oxide interface (Δ*E* = −0.33 eV)^3^. On Cu(111), H_2_ dissociation is thermodynamically and kinetically (Δ*E* = −0.39 eV; *E*_a_ = 0.53 eV) more favorable than that on the ZnO–ZrO_2_ and Cu/oxide interfaces. Therefore, the presence of Cu is necessary to allow the facile formation of *H at the Cu/oxide interface under reaction conditions, which facilitates the subsequent hydrogenation processes by providing the surface *H species. This is consistent with the high pressure in situ DRIFTS (see Fig. 2 and Supplementary Fig. 12) results that CH_3_OH is detected over the CZZ sample but not over the ZnO–ZrO_2_ sample.

## Discussion

For the Cu/ZnO system, the Cu–ZnO interface or the Cu–Zn surface alloy are considered as the active sites for CO_2_ hydrogenation to methanol^3,5–7,24,28,29^. In the case of Cu/ZrO_2_, the Cu–ZrO_2_ interface play a very important role for methanol formation^10,26,27,33^. In both the binary catalysts, the catalytic activity is determined by the Cu–ZnO or Cu–ZrO_2_ interaction that is closely related to the physicochemical features (e.g., Cu particle size and surface area of Cu) of Cu spices. As shown in the comparison of the catalytic activity of Cu/ZnO, Cu–ZrO_2_, and Cu–ZnO–ZrO_2_ in Supplementary Fig. 13, the Cu–ZnO–ZrO_2_ ternary catalyst exhibits much higher methanol yield than either Cu–ZnO or Cu–ZrO_2_ even though it shows a lower surface area of Cu (*S*_Cu_) than the Cu/ZnO catalyst, suggesting that the ZnO–ZrO_2_ interaction should also play an important role in the Cu–ZnO–ZrO_2_ catalyst for CO_2_ hydrogenation. Combining the results of XPS (Supplementary Fig. 17) and CO_2_-TPD (Fig. 4d), it can be concluded that the ZnO–ZrO_2_ interaction promotes the formation of oxygen vacancies, which should be the active sites for CO_2_ adsorption. The in situ DRIFTS (Fig. 3 and Supplementary Fig. 12) experiments reveal that the ZnO–ZrO_2_ interface is crucial for the transformation of carbonate to formate during CO_2_ hydrogenation. However, no surface methoxy, which is a crucial intermediate species for methanol synthesis, is detected on the ZnO–ZrO_2_ catalyst (see Fig. 4a–c), while it is abundant on the Cu–ZnO–ZrO_2_ catalysts (see Supplementary Figs. 5 and 9). These results indicate that the presence of Cu is necessary for the formate hydrogenation to methoxy in methanol synthesis from CO_2_ + H_2_. It is reasonable to propose that, in the Cu–ZnO–ZrO_2_ system, the ZnO–ZrO_2_ interaction contributes to the adsorption of CO_2_ and binds the formate intermediate, and the interaction of Cu with the ZnO–ZrO_2_ support is responsible for the dissociative adsorption of hydrogen and the subsequent hydrogenation of carbonaceous intermediate species (e.g., formate and methoxy) to methanol. Supplementary Fig. 27 shows an illustration to emphasize on the role of Cu, ZnO, and ZrO_2_ in the ternary interaction and a full discussion is also provided.

Both the in situ DRIFTS experiments (Figs. 3 and 4) and DFT calculations (Fig. 5) suggest that CO_2_ adsorption and conversion to surface species (e.g., formate) and methanol strongly rely on the ZnO/ZrO_2_ interaction, which promotes the *HCOO activations, the rate-limiting step at the Cu/ZnO interface. Such promoting effect can be further enhanced by reducing the particle size of ZnO (Fig. 2 and Supplementary Fig. 8). The DFT results suggest that the presence of Cu at the ZnO–ZrO_2_ interface is necessary to provide *H and allows the conversion of the carbonate species to formate, methoxy and eventually methanol, as observed by in situ DRIFT under the activity test condition (Fig. 2) or during the switching from CO_2_ to H_2_ (Supplementary Fig. 5). Overall, the combination of in situ characterization and DFT calculations enables the identification of active sites and reaction intermediates for the CZZ catalyst during CO_2_ hydrogenation and highlights the importance of the strong interplay among Cu, ZnO, and ZrO_2_ in promoting the CO_2_ conversion and methanol selectivity.

In summary, by designing different Cu–ZnO–ZrO_2_ (CZZ) catalysts and performing in situ experiments and DFT calcinations, we identified the different functions of the ZnO–ZrO_2_ interface and Cu species in the catalytic CO_2_ hydrogenation to methanol. The obtained catalysts show very high activity (18.2% of conversion and 80.2% of selectivity obtained at 493 K and 3.0 MPa) for methanol generation. The formate pathway is identified for the CO_2_ hydrogeneration process over the CZZ catalysts. ZnO–ZrO_2_ binary oxides show much higher ability for CO_2_ adsorption and the hydrogenation of carbonate species to reactive intermediates (formate and methoxy) than the Cu–ZnO or Cu–ZrO_2_ systems. DFT calculations show that the ZnO/ZrO_2_ interface is the active site for CO_2_ adsorption and conversion, in particular for *HCOO activation, which is the rate-limiting step at the Cu/ZnO interface during CO_2_ hydrogenation. The presence of Cu^0^ is also necessary to allow the formation of *H under reaction conditions, while tuning the interaction between ZnO and ZrO_2_ can affect the formation and evolution of the surface species, therefore controlling the catalytic performance. The findings proposed in this work would enrich the knowledge in understanding the fundamental features of the CZZ ternary CO_2_ hydrogenation catalysts and be helpful for designing complex catalysts with multiple active components.

## Methods

### 3DOM catalyst preparation

3DOM catalysts were prepared by a colloidal crystal template method. First, uniform monodisperse poly methyl methacrylate (PMMA) spheres were synthesized as template via an emulsifier-free emulsion polymerization route^45^. After that, the required amounts of Cu(NO_3_)_2_·3H_2_O, Zn(NO_3_)_2_·6H_2_O, and Zr(NO_3_)_4_·5H_2_O were dissolved in 20 mL of deionized water to form a transparent solution (5 mol/L, Cu/Zn/Zr molar ratio = 5:2:3), and then 10 g of citric acid (C_6_H_8_O_7_·H_2_O) was added into the solution and dissolved at 333 K under stirring for 1 h. Then, 10 mL of ethylene glycol (EG) was added into the solution under stirring for 10 min. Subsequently, the dried PMMA templates were soaked in the precursor solutions for 6 h. After being filtered, the precursors were dried at 333 K for 12 h. Finally, the precursors were calcined at 723 K for 6 h with a ramp rate of 1, 2, 4, and 8 K/min under air, respectively. The obtained samples ware labeled as M-CZZ(16), M-CZZ(19), M-CZZ(24), and M-CZZ(36).

Cu–ZnO (molar ratio of Cu/Zn = 5:2), Cu–ZrO_2_ (molar ratio of Cu/Zr = 7:3), and ZnO–ZrO_2_ (molar ratio of Zn/Zr = 7:3) samples were prepared by a co-precipitation method. The required amounts of Cu(NO_3_)_2_·3H_2_O, Zn(NO_3_)_2_·6H_2_O, and/or Zr(NO_3_)_4_·5H_2_O were dissolved in 20 mL of deionized water to form a transparent solution (5 mol/L). Then, the transparent solution and NH_3_·H_2_O (2.5 wt.%) was added dropwise into 200 mL of deionized water, simultaneously, controlling the pH at ~6.5. After aging for 1 h, the precursor was filtered and washed with deionized water. The precursors was then dried at 333 K for 12 h and further calcined at 723 K for 3 h with a ramp rate of 2 K/min.

The ZnO–ZrO_2_ (molar ratio of Zn/Zr = 7:3) catalysts with different particle size of ZnO were also prepared by a co-precipitation method using different precipitating agent. The catalyst precipitated with sodium carbonate and calcined at 673 K for 6 h with a ramp rate of 1 K/min under air was labeled as ZnO(15)–ZrO_2_. The catalyst precipitated with sodium carbonate and calcined at 723 K for 6 h with a ramp rate of 1 K/min under air was labeled as ZnO(21)–ZrO_2_. The catalyst precipitated with NH_3_·H_2_O (2.5 wt.%) and calcined at 723 K for 6 h with a ramp rate of 2 K/min under air was labeled as ZnO(42)–ZrO_2_.

### Preparation of the nonporous samples

Conventional catalysts were prepared by a co-precipitation method. The required amounts of Cu(NO_3_)_2_·3H_2_O, Zn(NO_3_)_2_·6H_2_O, and Zr(NO_3_)_4_·5H_2_O were dissolved in 20 mL of deionized water to form a transparent solution (5 mol/L, Cu/Zn/Zr molar ratio = 5:2:3). Then, the transparent solution and NH_3_·H_2_O (2.5 wt.%) was added dropwise into 200 mL of deionized water, simultaneously, controlling the the pH at ~6.5. After aging for 1 h, the precursor was filtered and washed with deionized water. The precursors was then dried at 333 K for 12 h and further calcined at 723 K for 3 h with a ramp rate of 2 and 5 K/min, and the obtained samples are labeled as N-CZZ(25) and N-CZZ(31), respectively. When the precipitating agent was changed from sodium carbonate (0.5 mol/L) to NH_3_·H_2_O (2.5 wt.%) and calcining the precursor at 723 K for 3 h with a ramp rate of 5 K/min, the obtained sample was labeled as N-CZZ(43).

### Characterization

The specific surface area of the catalysts was calculated according to the BET method using the N_2_ adsorption isotherm at 77 K obtained on a Quantachrome Autosorb-iQ instrument. The crystal phases of the prepared catalysts were identified using a powder X-ray diffractometer (Rigaku D/max-R) with Cu Ka radiation (*λ* = 0.15406 nm). The X-ray tube was operated at 40 kV and 40 mA. The XRD patterns were recorded for 2*θ* values ranging from 20° to 80° at a scanning rate of 2°/min. The morphology of the catalysts was observed by the scanning electron microscopy (SEM) and transmission electron microscopy (TEM) technology. The SEM measurement was performed on a Nova NanoSEM 450 instrument using an accelerating voltage of 2000 V–30 kV. The SEM samples were dusted on an adhesive conductive carbon belt attached to a copper disk and coated with 2–3 nm Pt prior to the measurement. For TEM characterization, a Tecnai G^2^ TF30 S-Twin microscope was used operating at 300 kV. The specimens were crushed into powder and immersed in a small volume of ethanol. After sonicating the mixture for 10 min, a droplet of the suspension was allowed to dry on a holey carbon/Formvar-coated copper TEM grid.

XPS dates were obtained with a PHI 5000 Versaprobe II system equipped with a monochromatic Al-Ka X-ray source. The reduced samples were placed on stainless steel sample-holders were transferred to the XPS pre-chamber under inert atmosphere and stayed there for 12 h in a vacuum atmosphere. The spectra were recorded after purging the samples at ambient temperature under vacuum (residual pressure < 10^−7^ Pa). The C 1s signal at 284.8 eV was used as an internal standard for calibration of the XPS signals.

H_2_ temperature-programmed reduction (H_2_-TPR) was performed on a ChemBET Pulsar & TPR/TPD apparatus (Quantachrome Instruments) with a thermal conductivity detector (TCD). Prior to each experiment, the sample (50 mg) was pre-treated in flowing pure He (30 mL/min) at 573 K for 1 h and cooled to room temperature (RT). Thereafter, the temperature was increased at a rate of 10 K/min to 673 K in flowing 10% H_2_/Ar (30 mL/min). The metallic copper surface area (*S*_Cu_) was measured using N_2_O decomposition method. The catalyst (50 mg) was first reduced with 10% H_2_/Ar (30 mL/min) at 573 K for 1 h followed by purging with He (30 mL/min) for 30 min and cooling to 333 K. Then, a flow of 10% N_2_O/He (30 mL/min) gas was fed into the reactor for 1 h. TPR measurement was subsequently performed under a 10% H_2_/Ar flow (30 mL/min) to 573 K with a ramp rate of 10 K/min. The copper surface area was calculated on base of Eq. (1) by assuming that the copper crystallites are spherical.

1 $$S_{\mathrm{Cu}}\left( {\mathrm{m}}^{2}/{\mathrm{g}}_{\mathrm{cat}} \right) = \left[ 200 \left( {\mathrm{Mol}} \hskip 3pt {\mathrm{H}}_{2} \right) \times \left( {\mathrm{SF}} \right) \times \left( N_{\mathrm{A}} \right) \right] / \left( {\mathrm{SD}}_{\mathrm{Cu}} \right)$$

2 $$D_{\mathrm{Cu}} ( \% ) = [{\mathrm{the}} \hskip 3pt {\mathrm{amount}} \hskip 3pt {\mathrm{of}} \hskip 3pt {\mathrm{exposed}} \hskip 3pt {\mathrm{copper}}] / [{\mathrm{the}} \hskip 3pt {\mathrm{total}} \hskip 3pt {\mathrm{amount}} \hskip 3pt {\mathrm{of}} \hskip 3pt {\mathrm{copper}} \hskip 3pt {\mathrm{atoms}}]$$

Where Mol H_2_ is the amount of H_2_ consumed during the TPR step per unit mass of the catalyst (mol H_2_/g_cat_), SF is the stoichiometric factor (2.0), *N*_A_ is Avogadro’s number (6.022 × 10^23^ atoms/mol) and SD_Cu_ is the copper surface density (1.47 × 10^19^ atoms/m^2^).

3 $${\mathrm{TOF}}_{\mathrm{Cu}} = \frac{P_{{\mathrm{CH}}_{3}{\mathrm{OH}}}\, \times M_{\mathrm{Cu}}}{3600 \times 1000\, \times \left( D\; \times 0.01 \right)\times X_{\mathrm{Cu}}}$$

Where $P_{{\mathrm{CH}}_{3}{\mathrm{OH}}}$, *M*_Cu_, *D*, *X*_Cu_ are CH_3_OH productivity (expressed in mol h^−1^ kg^−1^), the copper molecular weight, the copper dispersion (expressed in %), the average mass fraction of Cu in fresh catalyst, respectively.

CO temperature-programmed reduction (CO-TPR) was performed on a the CATLAB catalyst characterization system (Hiden Analytical Co., England). Prior to each experiment, the sample (50 mg) was pre-treated in flowing pure He (30 mL/min) at 573 K for 1 h and then cooled to RT. Thereafter, the temperature was increased at a rate of 10 K/min to 973 K in flowing 10% CO/Ar (30 mL/min). The gas was analyzed by an online mass spectrometer (MS).

CO_2_ temperature-programmed desorption (CO_2_-TPD) was performed on a ChemBET Pulsar & TPR/TPD apparatus (Quantachrome Instruments) equipped with a TCD. Prior to each experiment, the sample (50 mg) was pre-treated in flowing pure He (30 mL/min) at 573 K for 1 h and then cooled to RT. Thereafter, the temperature was increased at a rate of 10 K/min to 573 K in flowing 10% H_2_/Ar (30 mL/min) and holding at 573 K for 30 min. Thereafter, the pre-treated sample was exposed to CO_2_ (30 mL/min) and He (30 mL/min) at RT for 30 min, respectively. After that, the temperature was increased at a rate of 10 K/min to 873 K in flowing He (30 mL/min).

In situ DRIFTS measurements were performed on an FTIR spectrometer (vertex 70, Bruker, Germany) equipped with a liquid nitrogen N_2_ cooled mercury–cadmium–telluride (MCT) detector. The scans were collected from 4000 to 600 cm^−1^ at a resolution of 4 cm^−1^. The catalyst powders were placed in a high-pressure (0–10 MPa) DRIFTS cell (HC-900, Pike Technologies) equipped with ZnSe windows. To remove the possible residual surface species prior to testing, each sample was heated at 573 K for 2 h in a 40 mL/min flow of He. Then, the sample was cooled to 323 K and switching feed to 10% H_2_/He mixture at a flow rate of 40 mL/min while increasing the temperature to 573 K for 1 h. After that, the sample was flushed with He (40 mL/min) for 1 h and cooled to 493 K prior to sample testing. The background subtractions were executed over different samples for testing in a 40 mL/min He under different reaction condition (atmospheric pressure or 3 MPa). After that, the reaction gases were switched into the reaction chamber, the evolutions of functional groups on samples surfaces were recorded by FTIR spectrometer.

### Activity test

The activity test for CO_2_ hydrogenation to methanol were performed in a high-pressure fixed-bed flow stainless steel reactor. One gram of catalyst was diluted with quartz sand (both in 20–40 mesh), and then packed into the stainless steel tubular reactor. Prior to the catalytic measurements, the catalyst was reduced in a stream of 10% H_2_/N_2_ at 573 K for 6 h under atmospheric pressure. Then, the temperature was cooled to 323 K, and the reductive gas was replaced by the reaction gas (24.4% CO_2_ and 75.6% H_2_). The reaction was performed with a pressure of 3.0 MPa, reaction gases flow rate is 100 mL/min, and the temperature at 493 K. Each reaction was conducted under these conditions for 16 h. The reactants and products flowing out in the reactors were passed through the gas/liquid separator connected to a heat exchanger (273 K) and then analyzed by an online gas chromatographer (GC, Agilent Technologies 6890A) equipped with a TCD and a flame ionization detector (FID). The CO_2_ conversion and CH_3_OH selectivity were obtained from the GC data.

### Computational methods

Spin polarized density functional theory (DFT)^46,47^ calculations were performed using the Vienna Ab-Initio Simulation Package (VASP) code.^48,49^ Projector augmented wave (PAW)^50^ potentials were used to describe the core electrons with the generalized gradient approximation (GGA) using PW91 functionals^51^. The Kohn–Sham one-electron wave functions were expanded by using a plane wave basis set with a kinetic energy cutoff of 400 eV.

The lattice parameters of bulk ZnO was calculated using a 12 × 12 × 8 Monkhorst^52^ pack meshes. Our calculated lattice parameters of *a* = *b* = 3.194 Å and *c*/*a* = 1.608 Å are similar to the previously calculated values of *a* = *b* = 3.159 and *c*/*a* = 1.608 using DFT^53^. The ZnO(110) surface was modeled using a six layer 3 × 3 surface slab. The ZrO_2_/ZnO interface was modeled by depositing a small ZrO_2_ cluster on ZnO(110) surface. Surface O atoms on ZrO_2_ cluster and ZnO(110) surface were hydroxylated to take into account of the H-spillover under the H_2_-rich conditions used for CO_2_ hydrogenation. The electronic structure of Zn in ZnO was treated in the DFT + *U*^54^ formalism with a *U* value of 7.5 eV^53^. The Brillouin zone of the ZnO(110) surface was sampled using the Γ-point. A 18 Å thick vacuum was added along the direction perpendicular to the surface in the initial slab model to avoid the artificial interactions between the slab and its periodic images. During geometry optimization, the atoms in the bottom three layers were fixed while all other atoms were allowed to relax until Hellman–Feynman force on each ion was smaller than 0.02 eV/Å. The binding energy (BE) of an adsorbate was calculated as follows:

$$\mathrm{BE} = {\it E} \left( {{\mathrm{slab + adsorbate}}} \right) - {\it E} \left( {{\mathrm{slab}}} \right) - {\it E} \left( {{\mathrm{adsorbate}}} \right)$$

where *E*(slab + adsorbate), *E*(slab), and *E*(adsorbate) are the total energies of the slab with adsorbate, clean slab, and adsorbate species in the gas phase, respectively.

The transition state of a chemical reaction was located using the climbing image nudged elastic band (CI-NEB) method implemented in VASP^55^. The activation energy (*E*_a_) of a chemical reaction is defined as the energy difference between the initial and transition states while the reaction energy (Δ*E*) is defined as the energy difference between the initial and final states.

## Supplementary information

Supplementary Information

Peer Review File

## Data Availability

All data are available from the authors on reasonable request.
