# Supplementary material for: Exploring the ternary interactions in Cu–ZnO–ZrO2 catalysts for efficient CO2 hydrogenation to methanol
Source: Nat Commun. 2019 Mar 11;10:1166. doi: 10.1038/s41467-019-09072-6 (PMC6411953; doi:10.1038/s41467-019-09072-6)
Supplement: Supplementary file 1 — Supplementary Information [file 41467_2019_9072_MOESM1_ESM.pdf]

Exploring the ternary interactions in Cu-ZnO-ZrO_2 catalysts for efficient CO_2 hydrogenation to methanol

Wang et al.

Supplementary Information

Table of Contents

Supplementary Figure 1..4

Supplementary Figure 2..6

Supplementary Figure 3..7

Supplementary Figure 4..8

Supplementary Figure 5..10

Supplementary Figure 6..11

Supplementary Figure 7..12

Supplementary Figure 8..13

Supplementary Figure 9..14

Supplementary Figure 10..15

Supplementary Figure 11..16

Supplementary Figure 12..17

Supplementary Figure 13..18

Supplementary Figure 14..19

Supplementary Figure 15..21

Supplementary Figure 16..22

Supplementary Figure 17..23

Supplementary Figure 18..25

Supplementary Figure 19..26

Supplementary Figure 20..27

Supplementary Figure 21..28

Supplementary Figure 22..29

Supplementary Figure 23..30

Supplementary Figure 24..31

Supplementary Figure 25..33

Supplementary Figure 26..34

Supplementary Figure 27..35

Supplementary Table 1...36

Supplementary Table 2...37

Supplementary Table 3...38

Supplementary Table 4...39

Supplementary Table 5...40

Supplementary Table 6...41

Supplementary Table 7...42

References……………...46

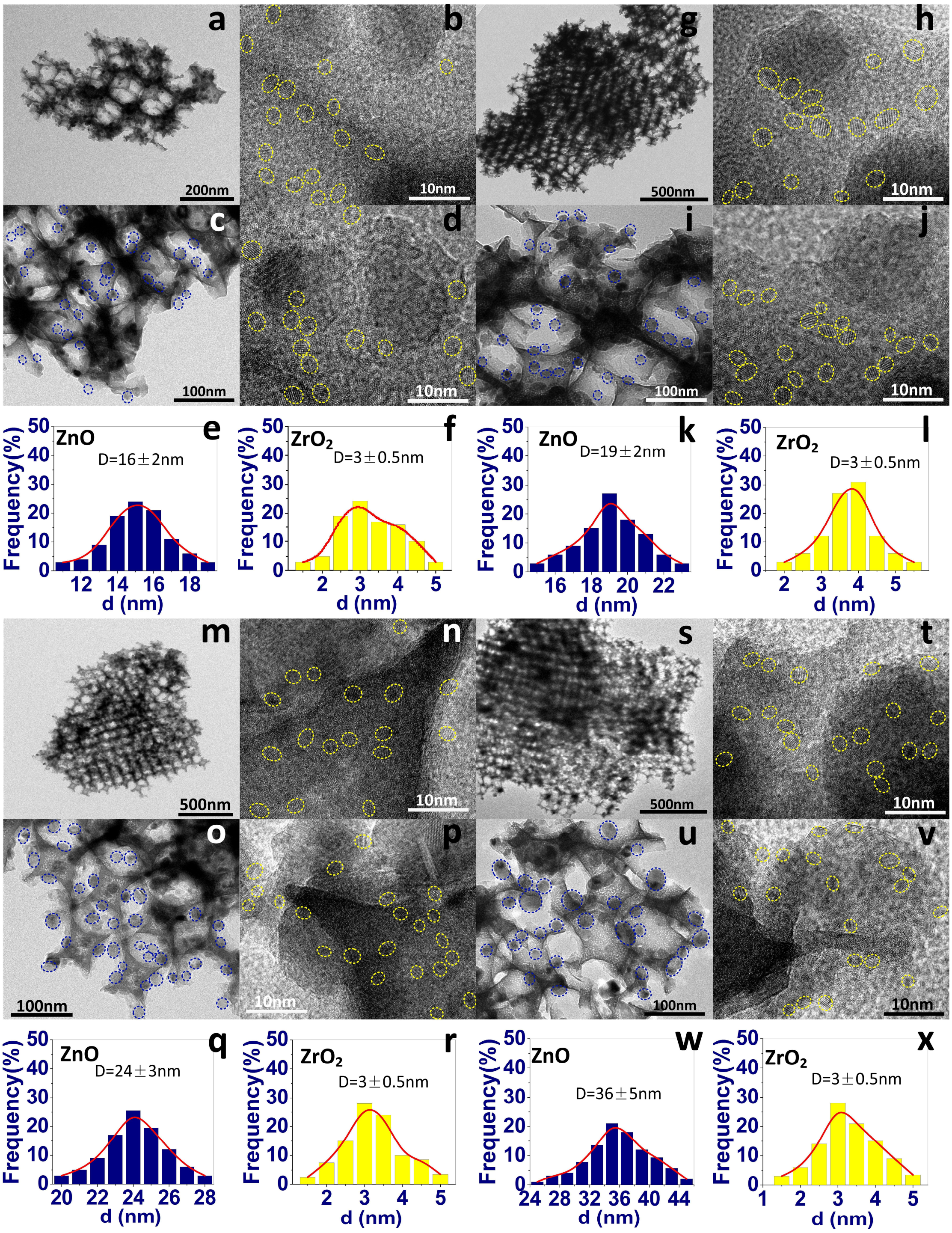

Supplementary Figure 1. TEM images and size distribution of ZnO and ZrO_2 particles in different 3DOM samples. The samples with different particle size of ZnO are obtained by calcining the precursor at 723 K for 3 h with different ramp rates in air. **a-f,** M-CZZ (16) sample prepared by a ramp rate of 1 K/ min. **g-l**, M-CZZ (19) sample prepared by a ramp rate of 2 K/min. **m-r**, M-CZZ (24) sample prepared by a ramp rate of 4 K/ min. **s-x,** M-CZZ (36) sample prepared

by a ramp rate of 8 K/ min. The average particle sizes of ZnO and ZrO_2 are obtained by statistical analysis of more than 300 particles of each oxide.

By controlling the ramp rate in the calculation process, the samples with different ZnO particle sizes (the average particle size of ZnO is 15.8, 19.1, 23.9 and 35.3 for the M-CZZ (16), M-CZZ (19), M-CZZ (24) and M-CZZ (35) samples, respectively) but similar ZrO_2 particle sizes (3-4 nm) are obtained. All the four samples show uniform macroporous structure.

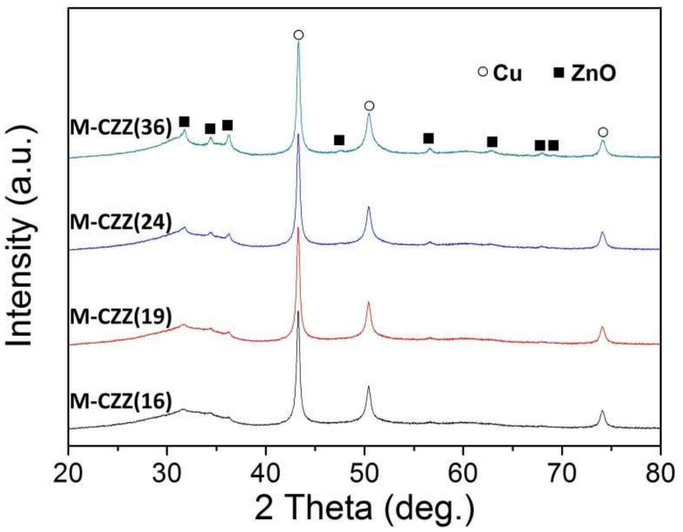

Supplementary Figure 2. XRD patterns of the reduced 3DOM samples.

The diffraction peaks of Cu were detected at 43.3°, 50.5° and 74.1° and the main diffraction peaks of ZnO are detected at 31.7°, 34.4° and 36.2°. The diffraction peaks of ZrO_2 are barely visible among all the catalysts, indicating the very small particle size or poor crystallization of ZrO_2.

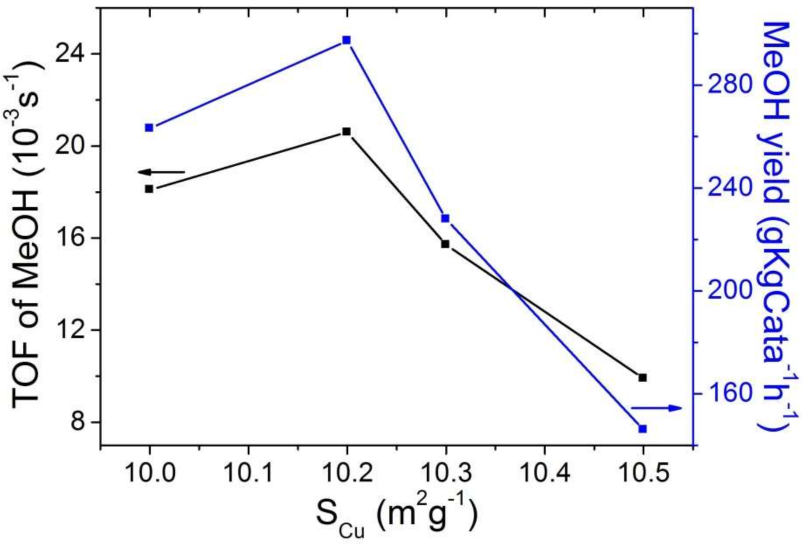

Supplementary Figure 3. The relationship between the TOF value/methanol yield and the Cu surface area (S_{Cu}) for the macroporous samples. It seems that the Cu surface area is not the determining factor for controlling the catalytic performance for methanol generation.

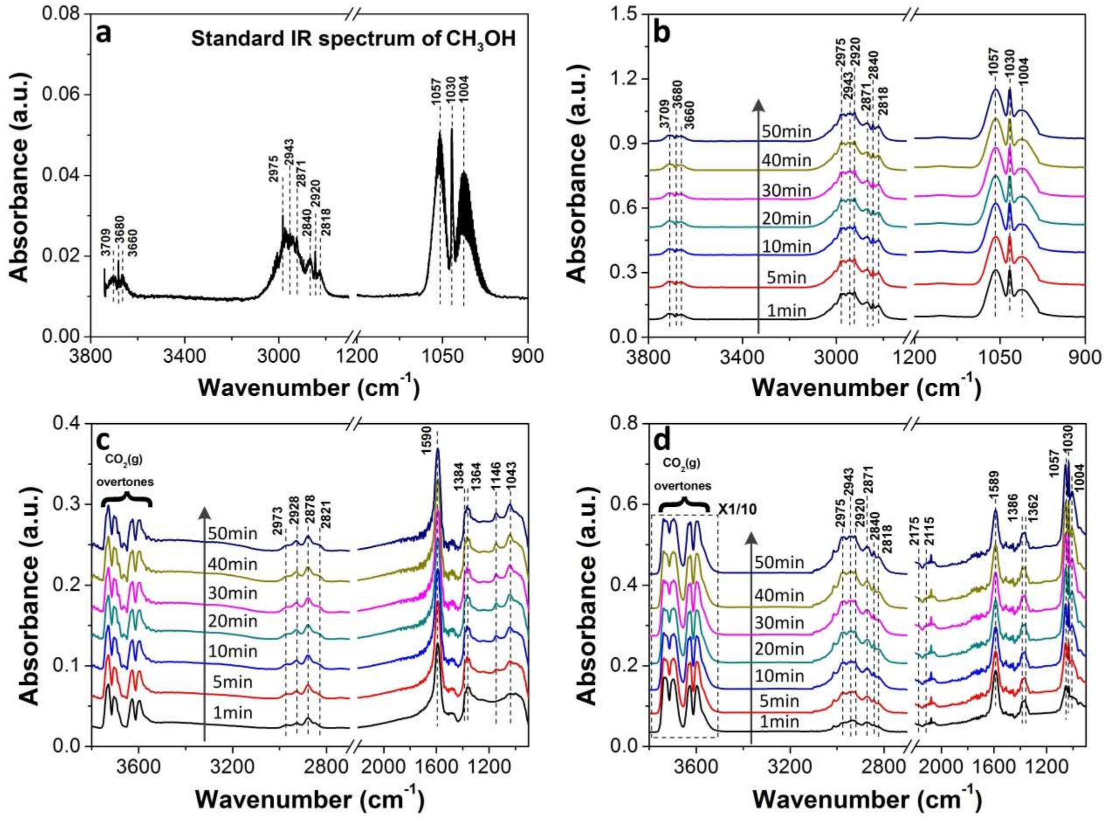

Supplementary Figure 4. Standard DRIFT spectrum of methanol (**a**). I*n situ* DRIFT spectra of methanol adsorption (**b**) on the M-CZZ (16) catalyst at 493 K. *In situ* DRIFT spectra of the CO_2 + H_2 reaction over the M-CZZ (16) sample under ambient pressure (**c**). *In situ* DRIFT spectra of the CO_2 + H_2 reaction over the M-CZZ (16) sample under realistic reaction condition (3MPa) (**d**)

Supplementary Figure 4a shows the standard DRIFT spectrum of methanol provided by BRUKER, and the *in situ* DRIFT spectra of methanol adsorption on the M-CZZ (16) catalyst at 493 K is presented in Supplementary Figure 4b. As can be seen, the vibrational bands at 1057, 1030 and 1004 cm^{-1} can be corresponding to the C-O stretch of methanol, and bands at 2975, 2943, 2920, 2840 and 2818 cm^{-1} are attributed to the C-H stretch of methanol. The O-H stretching mode of methanol is detected in the range of from 3600 to 3800 cm^{-1}. The infrared peaks position of methanol on M-CZZ (16) catalyst at 493 K are exactly the same with that of the

standard methanol DRIFT spectrum. The *in situ* DRIFT spectra of the CO_2 + H_2 reaction over the M-CZZ (16) catalyst under ambient pressure is showed in Supplementary Figure 4c.The bands for formate species are located at 2967, 2878, 1590, 1384, and 1364 cm^{-1}. The C-H (2928 and 2821 cm^{-1}) and C-O (1146 and 1043 cm^{-1}) stretching features attributed to the methoxy are also observed. It should be highlighted that the C-O stretching mode of methanol is detected at ca. 1055, 1030 and 1005, while it appears at ca. 1150 and 1050 for the methoxy. In this case, the band at ca. 1005 cm^{-1} could be used to identify the formation of methanol. On the other hand, the absence of O-H stretching mode of methanol in the *in situ* DRIFT experiments under 3MPa should be attributed to the overlapping of CO_2 bands in the range of 3600-3800 cm^{-1}.

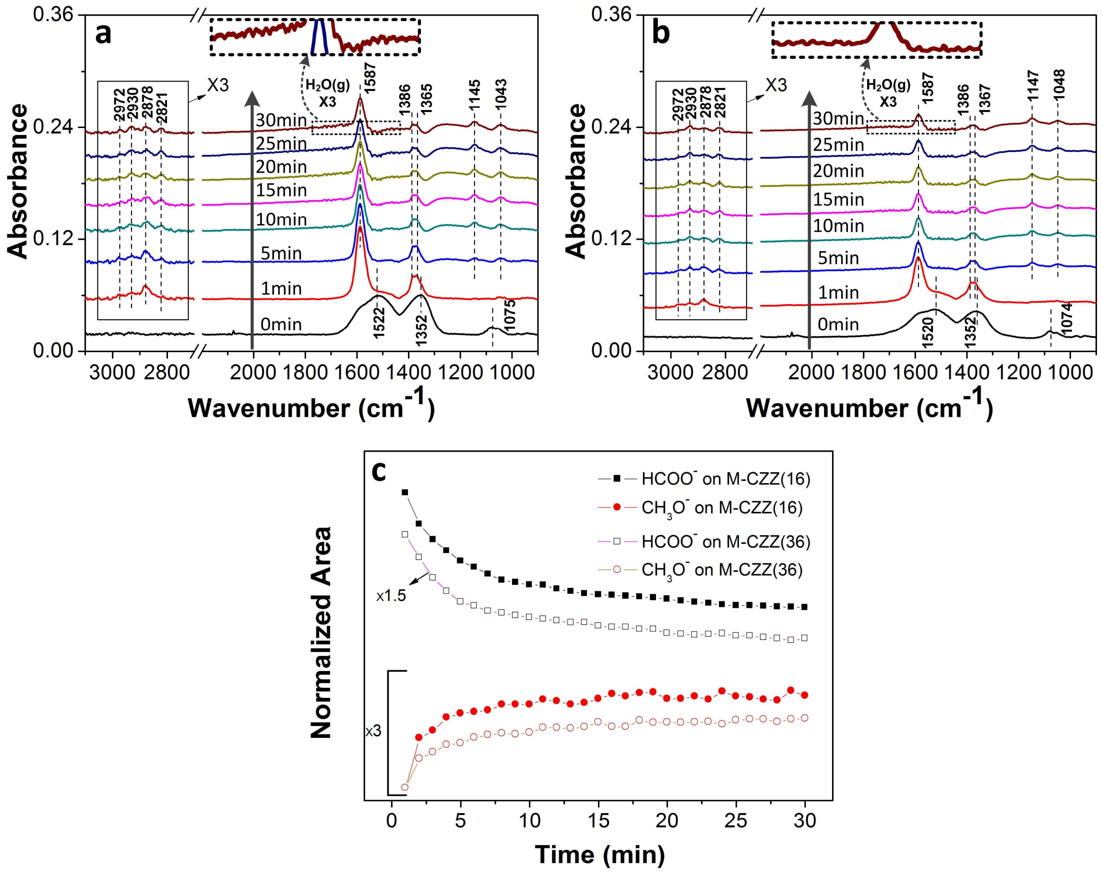

Supplementary Figure 5. Evolution of surface species in designed conditions over the different microporous samples. a and b, *In situ* DRIFT spectra over (**a**) M-CZZ (16) and (**b**) M-CZZ (36) catalysts when switching the CO_2 feed gas (CO_2 has been loaded into the camber for 10 min) to H_2 at 493 K and 0.1 MPa). **c**, Peak areas of generated intermediate species and methanol during the experiments: areas normalized to the values observed at the end of the transient. The M-CZZ (16) sample shows much higher ability for CO_2 adsorption (carbonate species at 1522 and 1352 cm^{-1}) and their further conversion to formate (2972, 2878, 1587, 1386, and 1365 cm^{-1}) and methoxy species (2930, 2821,1145 and 1043 cm^{-1}) than the M-CZZ (36) sample. This suggests that the particle size of ZnO affects the CO_2 adsorption and its further conversion.

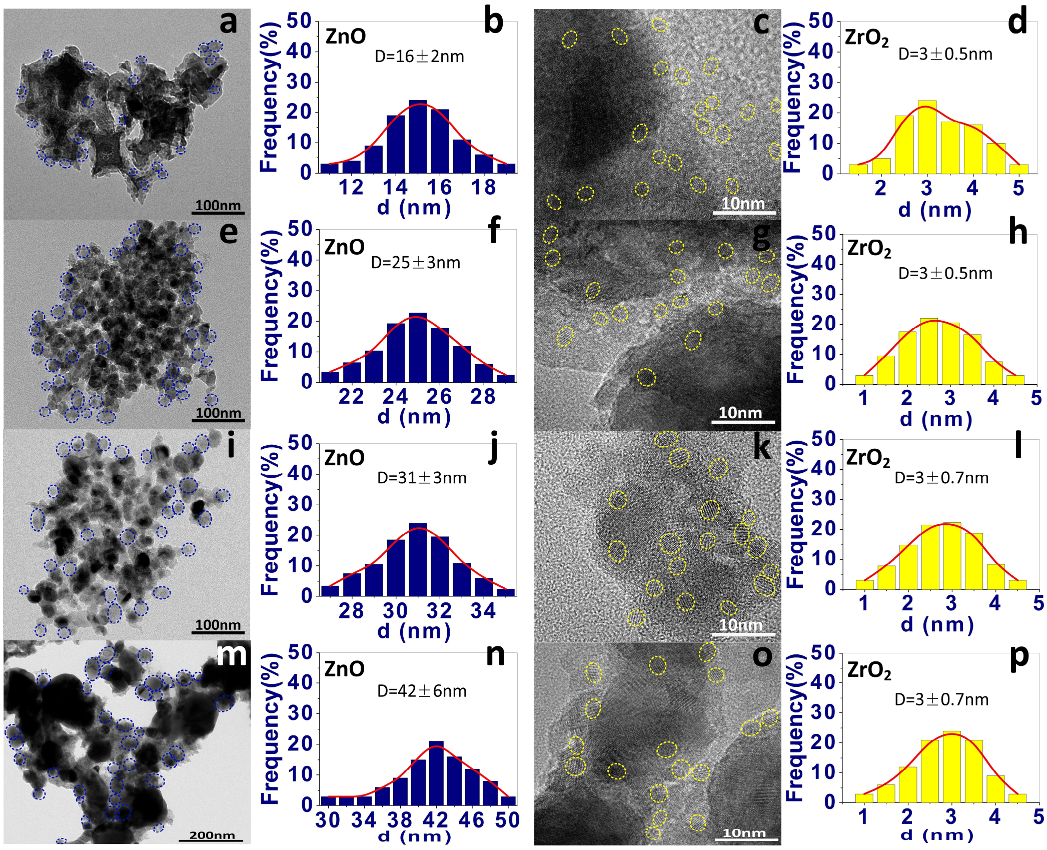

Supplementary Figure 6. TEM images and size distribution of ZnO and ZrO_2 particles in the nonporous samples. **a-d,** N-CZZ (16), which is obtained by grinding the M-CZZ (16) sample. **e-h**, N-CZZ (25), which is prepared by a co-precipitation method under the conditions: using sodium carbonate as precipitator, calcination at 723 K for 3 h in air with a ramp rate of 2 K/ min. **i-l**, N-CZZ (31), which is prepared by a co-precipitation method under the conditions: using sodium carbonate as precipitator, calcination at 723 K for 3 h in air with a ramp rate of 8 K/ min. **m-p**, N-CZZ (42), which is prepared by a co-precipitation method under the conditions: using ammonia as precipitator, calcination at 723 K for 3 h in air with a ramp rate of 2 K/ min. Cu particles agglomerate into some big islands (see the dark areas), which were sounded by the ZnO particles. ZrO_2 nanoparticles (3.5±1nm) are highly dispersed on the surface of ZnO and Cu matrix. The average particle size of ZnO is 15.8, 24.6, 31.4 and 43.2 for the N-CZZ (16), N-CZZ (25), N-CZZ (31) and N-CZZ (43) samples, respectively. The average ZrO_2 particle sizes (3-4 nm) are similar for all the four samples.

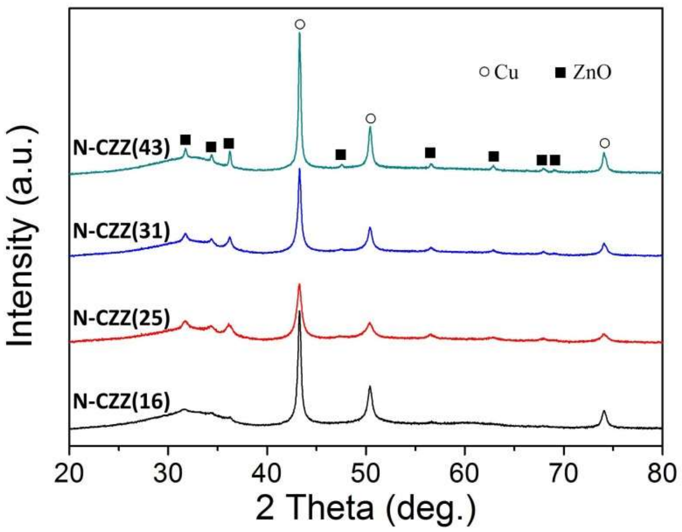

Supplementary Figure 7. XRD patterns of the reduced nonoporous samples.

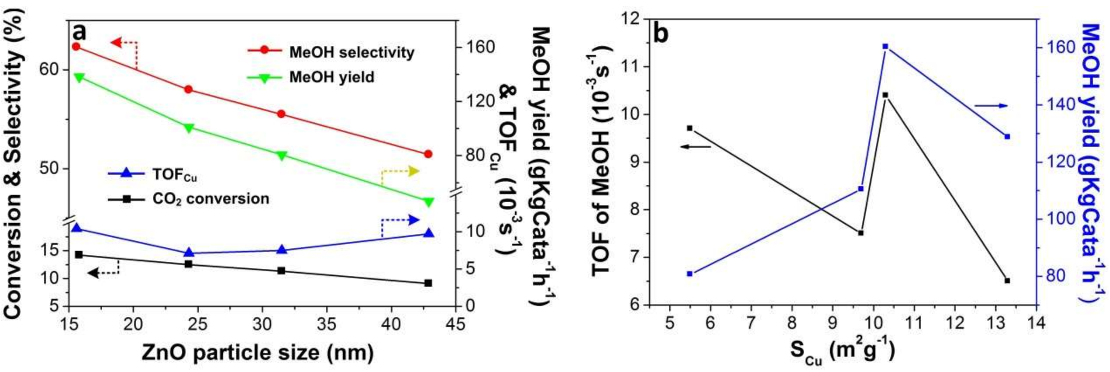

Supplementary Figure 8. Catalytic performance of the nonporous samples. **a**, CO_2 conversion, MeOH selectivity, MeOH yield and TOF values as a function of the ZnO_2 particle sizes in different nonporous catalysts. **b**, The relationship between the TOF value/methanol yield and the Cu surface area (S_{Cu}) for the macroporous samples.

Both the CO_2 conversion and methanol selectivity also obviously decrease with the increase of ZnO particle size. However, the TOF value increase when the ZnO particle size is higher than 25 nm. This can be attributed to the significantly reduced specific surface area of Cu (see Table S1), which is the other determining factor for calculating the TOF value. In addition, there is no serious dependence of TOF value on the metallic copper surface area, indicating that the Cu species may not be the determining factor for the catalytic performance.

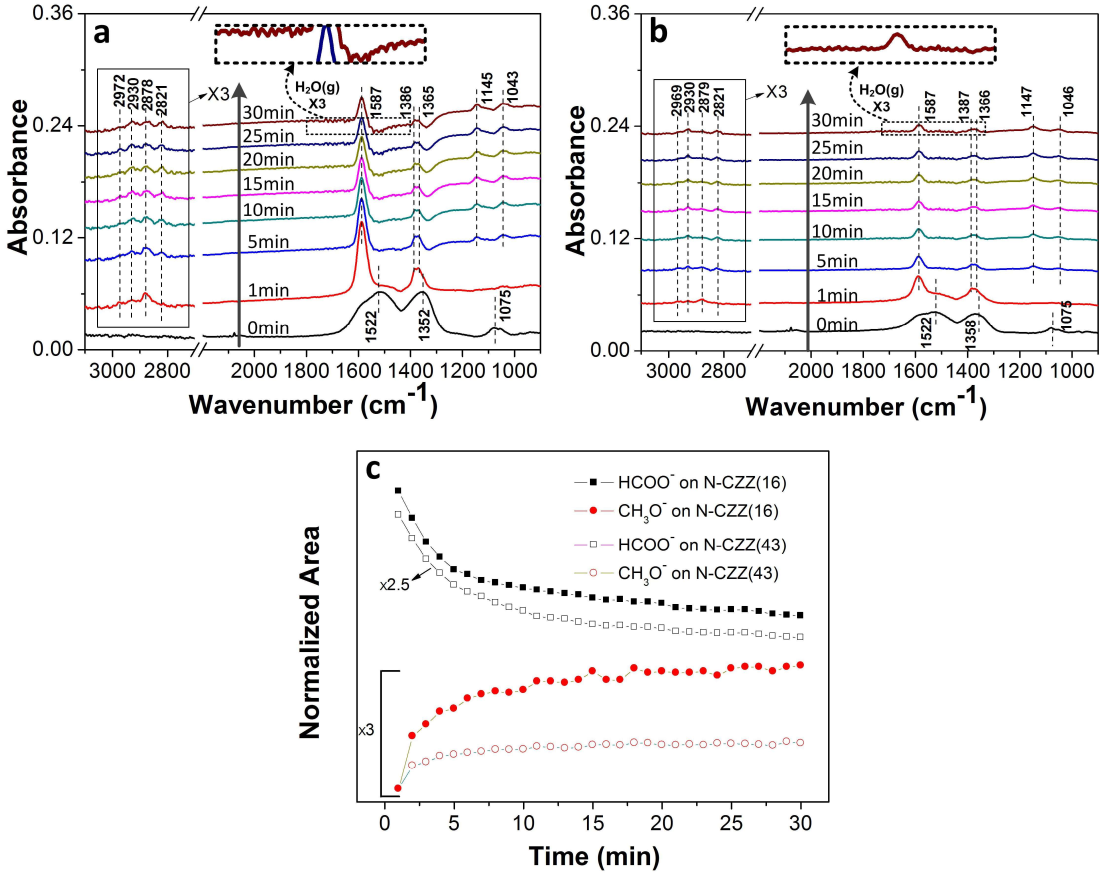

Supplementary Figure 9. Evolution of surface species in designed conditions over the different nonporous samples. **a** and **b**, *In situ* DRIFT spectra over (**a**) N-CZZ (16) and (**b**) N-CZZ (43) catalysts when switching the CO_2 feed gas (CO_2 has been loaded into the camber for 10 min) to H_2 at 493 K and 0.1 MPa). **c**, Peak areas of generated intermediate species and methanol during the experiments: areas normalized to the values observed at the end of the transient. Similar with that observed on the macroporous samples in Figure S4, smaller ZnO particle size results in higher intensity of carbonate species (1522 and 1352 cm^{-1}), formate (2972, 2878, 1587, 1386, and 1365 cm^{-1}) and methoxy species (2930, 2821,1145 and 1043 cm^{-1}) during the reactions.

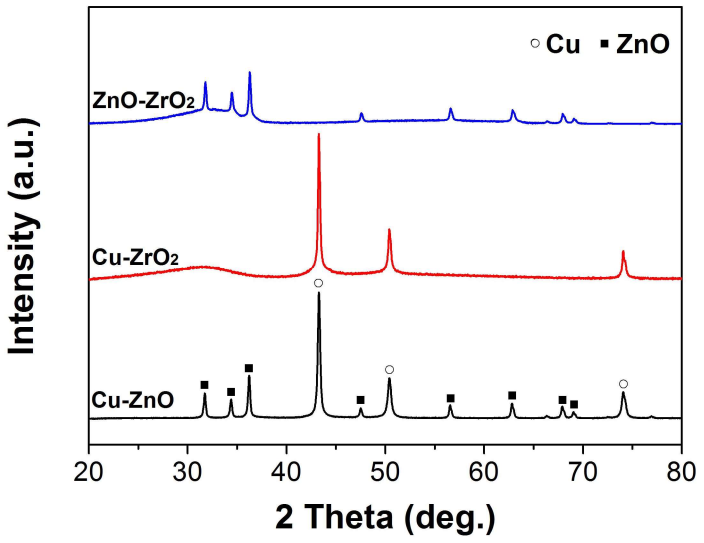

Supplementary Figure 10. XRD patterns of the Cu-ZnO, Cu-ZrO_2 and ZnO-ZrO_2 samples after reduction treatment by H_2.

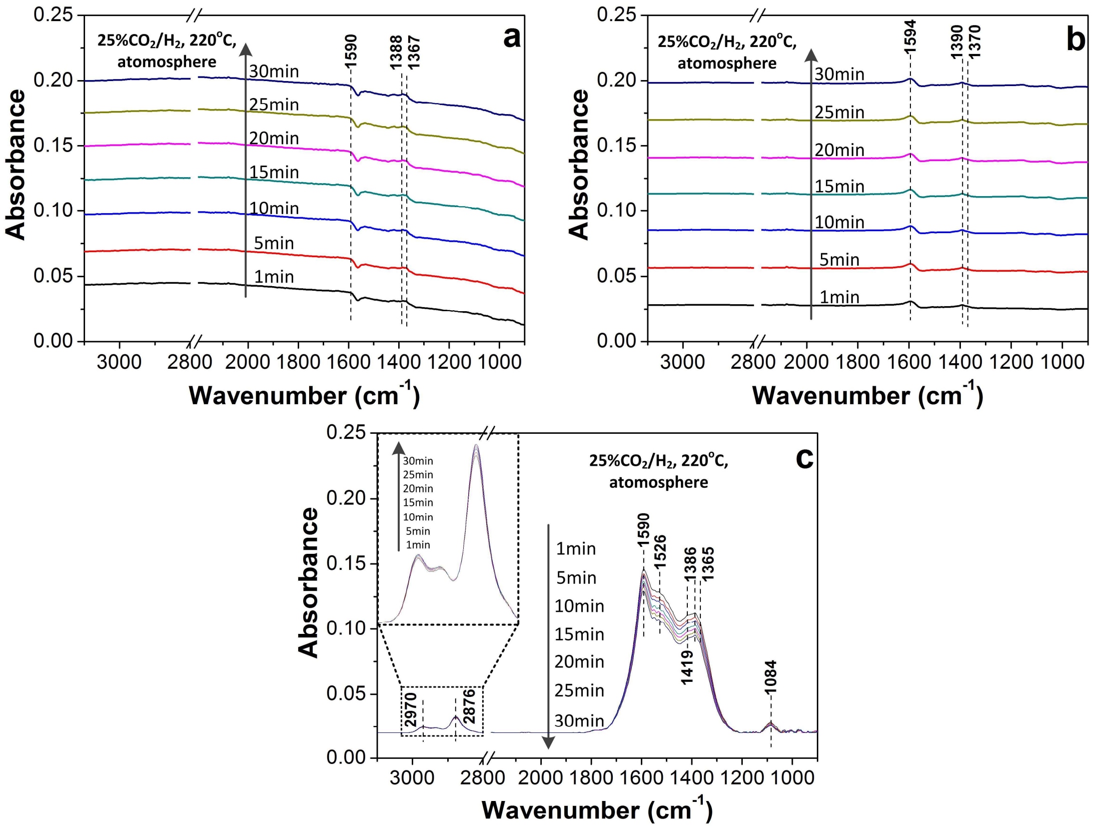

Supplementary Figure 11. *In situ* DRIFT spectra of Cu-ZnO (**a**), Cu-ZrO_2 (**b**) and ZnO-ZrO_2 (**c**) at 493 K after switching the feed gas from He to 25%CO_2/H_2 with a total rate of 40 mL/min under atmosphere pressure.

As switching to 25%CO_2/H_2, the very weak formate species (1590, 1388, 1367 cm^{-1}) were detected on Cu-ZnO and Cu-ZrO_2 samples. Interestingly, apparent carbonate species (1526, 1419, 1084 cm^{-1}) and formate species (2970, 2876, 1590, 1386, 1365 cm^{-1}) are observed on the ZnO-ZrO_2. This indicates that the ZnO-ZrO_2 interface should be the active sites for CO_2 adsorption.

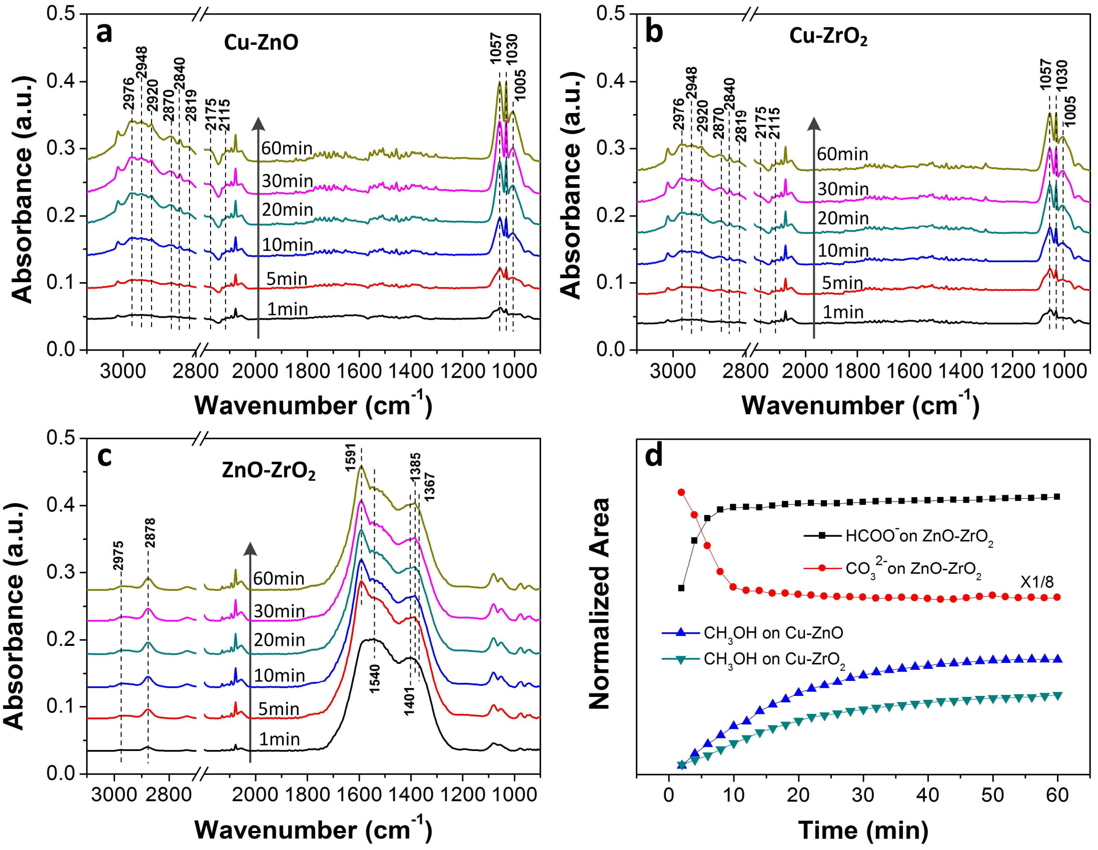

Supplementary Figure 12. *In situ* DRIFT spectra of Cu-ZnO (**a**), Cu-ZrO_2 (**b**) and ZnO-ZrO_2 (**c**) samples at 493 K after switching feed from He to 25%CO_2/H_2 with a total rate of 40 mL/min under 3MPa. d, **d**, Peak areas of generated intermediate species during the experiments: areas normalized to the values observed at the end of the transient.

As switching to 25%CO_2/H_2, the apparent carbonate species (1540, 1401 cm^{-1}) and formate species (2975, 2878, 1591, 1385 and 1367 cm^{-1}) are observed over the over binary ZnO-ZrO_2 at high pressure (3MPa). No obvious intermediate species are detected on the Cu-ZnO and Cu-ZrO_2 samples. Obvious signals at 1057, 1030 and 1005 cm^{-1} corresponding to the C-O stretch and bands at 2976, 2948, 2920, 2840 and 2819 cm^{-1} attributed to the C-H stretch are observed over the Cu-ZnO or Cu-ZrO_2 sample. This indicates the methanol formation over Cu-ZnO or Cu-ZrO_2 sample should not follow the formate pathway.

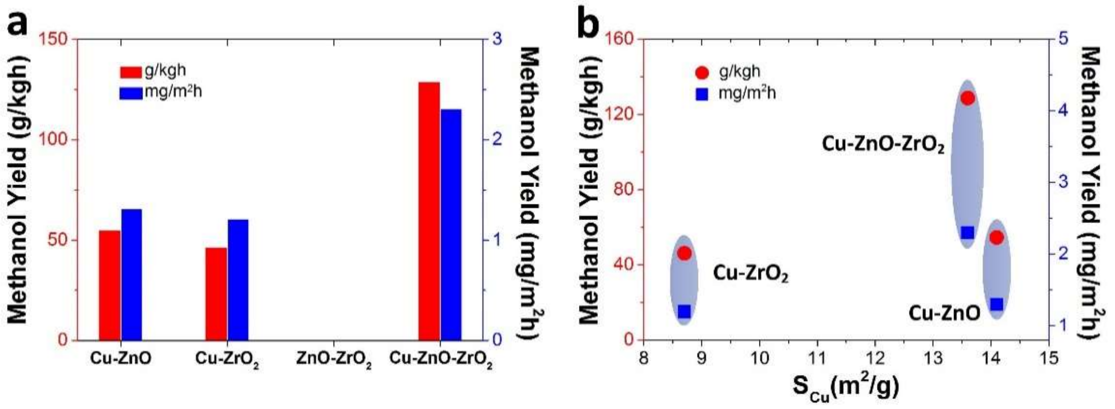

Supplementary Figure 13. (**a**) Methanol yields (normalized by specific surface area or normalized by weight) of Cu-ZnO, Cu-ZrO_2, ZnO-ZrO_2 and Cu-ZnO-ZrO_2 catalysts for CO_2 hydrogenation and (**b**) the relationship between S_{Cu} and methanol yield.

As shown in Supplementary Figure 13a, no methanol is detected over the ZnO-ZrO_2 catalyst for CO_2 hydrogenation, but both Cu-ZnO and Cu-ZrO_2 catalysts show good activity for this reaction. This indicates that the Cu/ZnO and Cu/ZrO_2 interactions are crucial for the methanol synthesis. On the other hand, the Cu-ZnO-ZrO_2 ternary catalyst exhibits much higher methanol yield than either Cu-ZnO or Cu-ZrO_2 even though it shows a lower surface area of Cu (S_{Cu}) than the Cu/ZnO catalyst (see Supplementary Figure 13b), suggesting that the ZnO-ZrO_2 interaction should also play an important role in the Cu-ZnO-ZrO_2 catalyst for CO_2 hydrogenation.

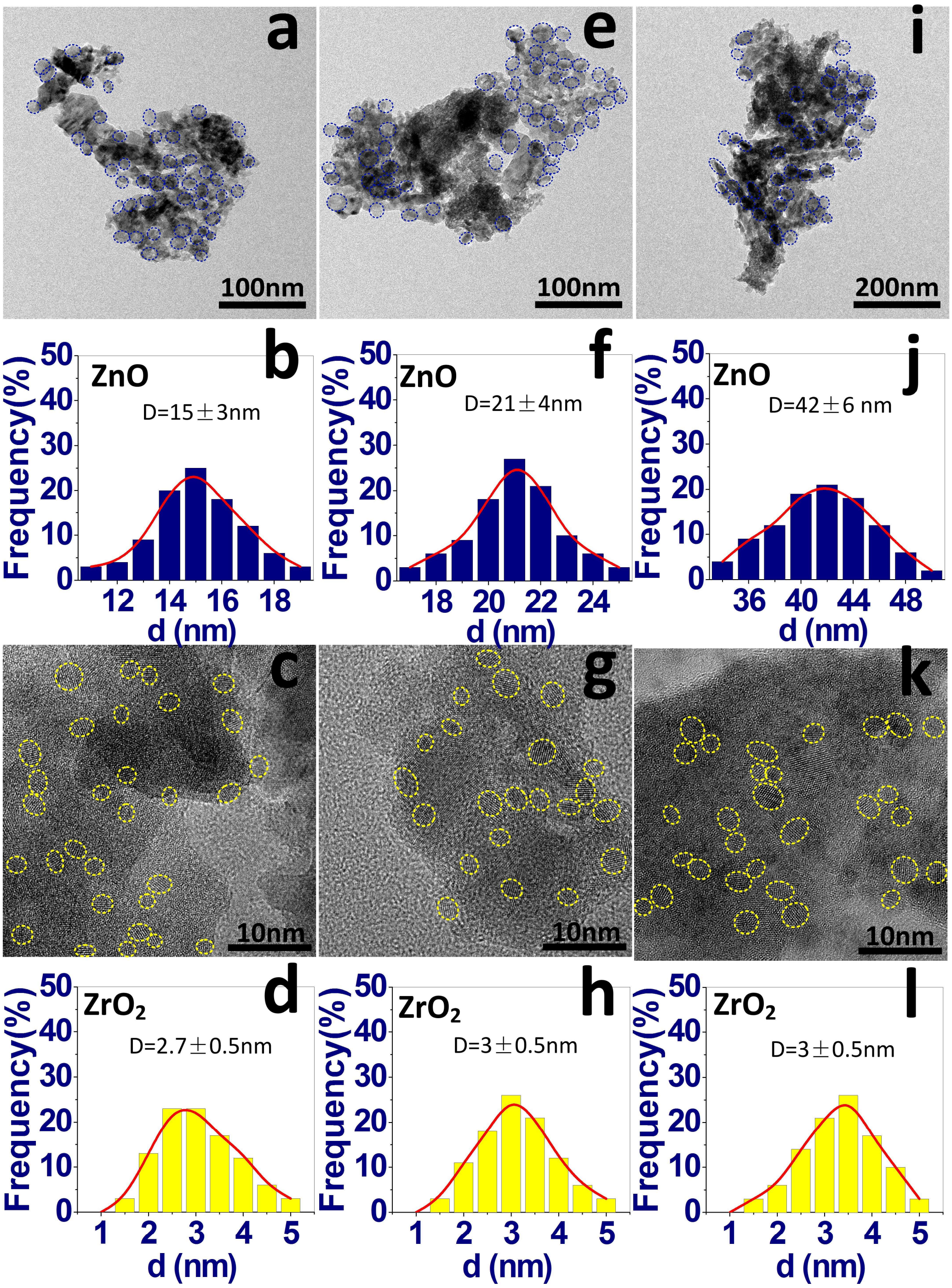

Supplementary Figure 14. TEM images and size distribution of ZnO particles in the different ZnO-ZrO_2 samples. **a-d**, ZnO (15)-ZrO_2 sample, which is prepared by a co-precipitation method under the conditions: using sodium carbonate as precipitator, calcinated at 673 K for 3 h with a ramp rate of 1 K/ min. **e-h**, ZnO (21)-ZrO_2 sample, which is prepared by a co-precipitation method under the conditions: using sodium carbonate as precipitator, calcinated at 723 K for 3 h

with a ramp rate of 1 K/ min. **i-l**, ZnO (42)-ZrO_2 sample, which is prepared by a co-precipitation method under the conditions: using ammonia as precipitator, calcined at 723 K for 3 h with a ramp rate of 2 K/ min. The average particle sizes of ZnO and ZrO_2 are obtained by statistical analysis of more than 300 particles of each oxide

The average particle size of ZnO is 15.2, 21.4 and 42.3 for the ZnO (15)-ZrO_2, ZnO (21)-ZrO_2 and ZnO (42)-ZrO_2 samples, respectively. The average ZrO_2 particle sizes (3-4 nm) are similar for all the four samples.

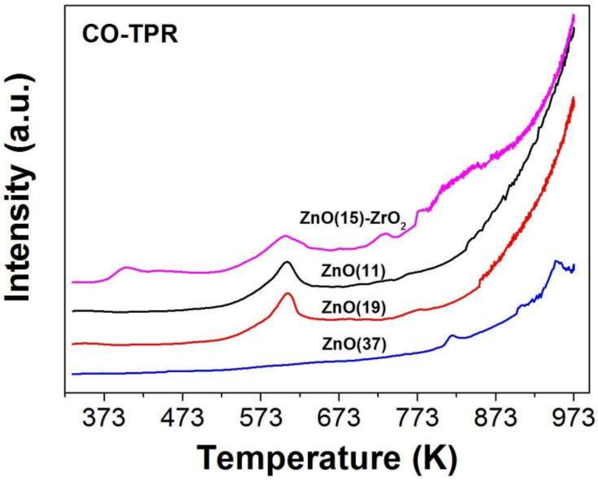

Supplementary Figure 15. CO-TPR of ZnO(15)-ZrO_2 sample and pure ZnO with different particle sizes.

In order to further understand the role of produced CO for Cu/ZnO/ZrO_2, we have performed CO-TPR measurements of ZnO with different particle sizes and the ZnO-ZrO_2 sample, and the signal of CO_2 is recorded using a mass spectrometer, as shown in Figure S15. It can be seen that a CO_2 peak is detected at 617 K for the pure ZnO sample with an average size of 11 or 19 nm. When the ZnO particle size increases to 37 nm, this low-temperature CO_2 peak disappears. This suggests that smaller particle size of ZnO could improve the reducibility. More importantly, there is an apparent CO_2 peak at very low temperatures (373~473 K, which is lower than the reaction temperature of 493 K for CO_2 hydrogenation in the present work) on the ZnO-ZrO_2 sample. The observation of this low-temperature peak indicates that the presence of CO in the reaction system may promote the formation of oxygen vacancies in the ZnO-ZrO_2 catalysts by reducing the ZnO.

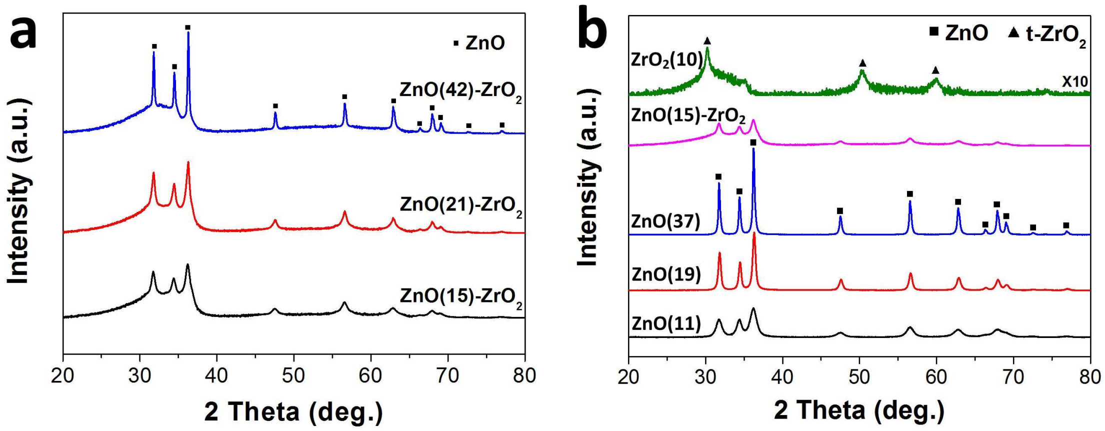

Supplementary Figure 16. XRD patterns of ZnO-ZrO_2 sample (**a**) with different average sizes (15, 21 and 42 nm), pure ZnO with different average sizes (11, 19 and 37 nm) and ZrO_2 with an average particle size of 10 nm (**b**).

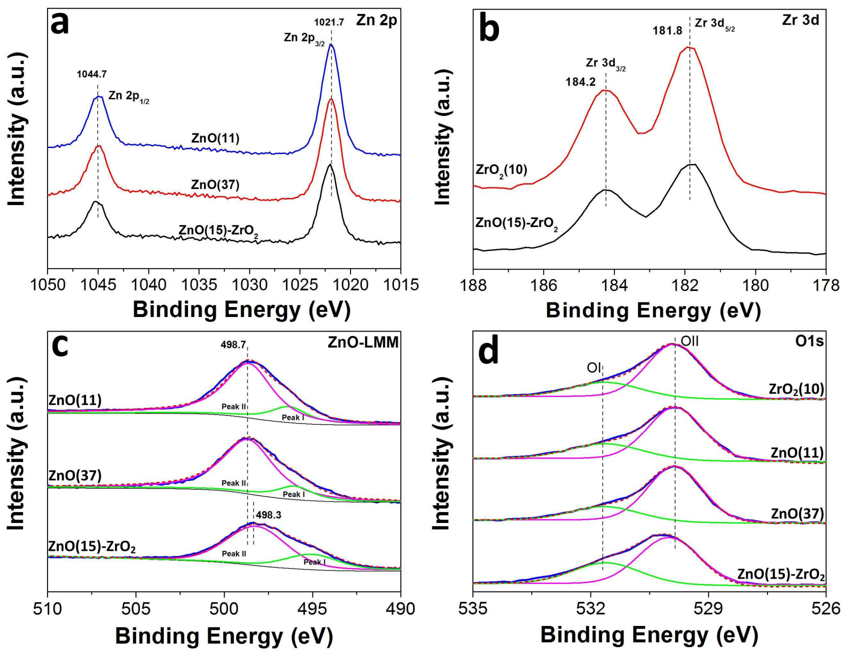

Supplementary Figure 17. XPS spectra of $ZnO-ZrO_2$ catalysts: Zn 2p (**a**), Zr 3d (**b**), Zn LMM (**c**), and O 1s (**d**).

To discuss the effect of ZnO particle size and the $ZnO-ZrO_2$ interaction on the formation of oxygen vacancy, XPS characterization was performed on the $ZnO-ZrO_2$ and ZnO samples with different particle sizes. According to the literature[1], the difference in the binding energy of Zn^0 and Zn^{2+} in the 2p XPS features is very small (about 0.3 eV). On the other hand, the change in the valence state from Zn^{2+} to Zn^0 is more obvious in the Auger measurements as a more pronounced 3 eV downward shift in the L_3MM Auger peak. As shown in Supplementary Figure 17c, the Zn L_3MM Auger peak was fitted to a main peak (Peak II) and a shoulder peak (Peak I). No obvious change is detected over the pure ZnO sample when changing the particle size. However, the peak for the $ZnO-ZrO_2$ sample is shifted by 0.4 eV to lower energy as compared to the pure ZnO. In addition, the shoulder peak is strongly enhanced for the

$ZnO-ZrO_2$ sample. As reported in the literature[1,2], the shift of the main peak and the change in the shoulder feature can be related to the formation of oxygen vacancies in both ZnO thin films and Cu-Zn alloys. Since there is no Cu in the $ZnO-ZrO_2$ sample, the observations in Supplementary Figure 17 suggest the generation of oxygen vacancy due to the strong $ZnO-ZrO_2$ interaction. The negative shift as compared to ZnO may be similar to the ZnO_x ($x < 2$) species, which is created by the interaction between Cu and ZnO[3,4] . The existence of oxygen vacancies is also reflected by the relative higher OI (surface oxygen)/OII (lattice oxygen) ratio in the O 1s peak for the $ZnO-ZrO_2$ sample (see Supplementary Figure 17d)[5]"

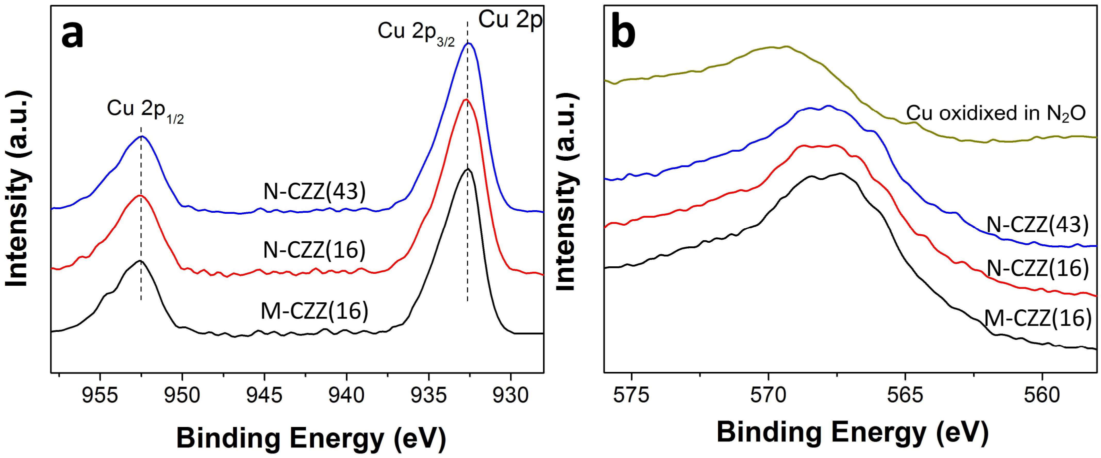

Supplementary Figure 18. XPS spectra of different CZZ samples after reaction: Cu 2p (**a**), Cu L3VV (**b**).

All the samples exhibited Cu $2p_{3/2}$ and Cu $2p_{1/2}$ main peaks with BEs values at approximately 932.6 and 952.4 eV, respectively, with a spin-orbit coupling energy of 19.8 eV. A shake-up satellite peak at approximately 942 eV was not detected, suggesting the absence of Cu^{2+} species[6]. It cannot be excluded the existence of Cu^{+}, since the BE of Cu^{+} generally overlaps with that of Cu^{0} in Cu 2p core level[6]. The results of Cu L_3VV Auger peaks are presented in Supplementary Figure 18b, which shows that the Cu nanoparticles are still zero valence after reaction[7].

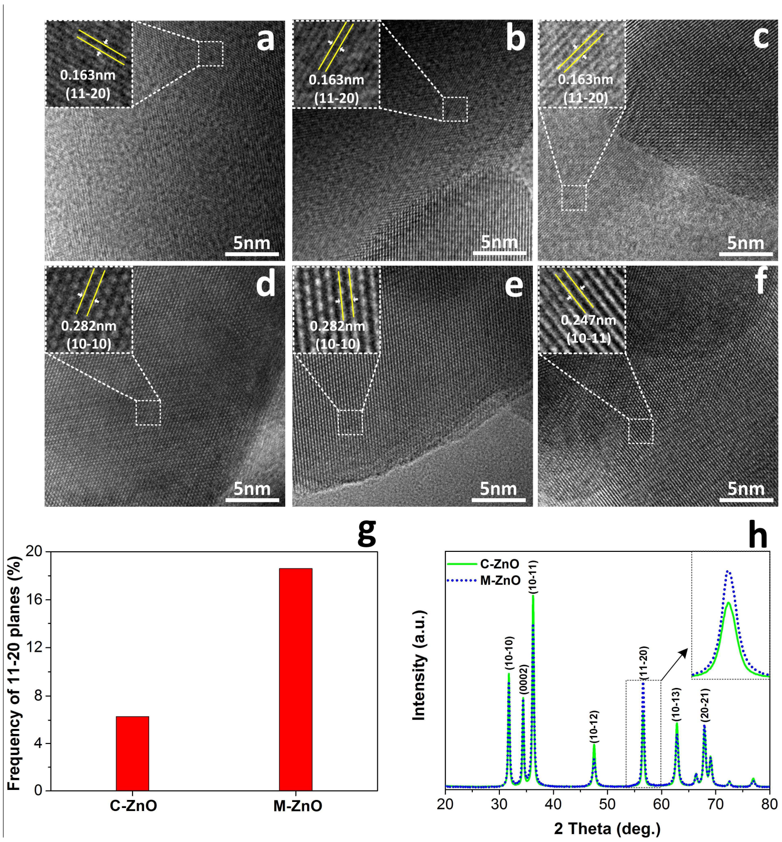

Supplementary Figure 19. HRTEM images of the M-ZnO (**a-c**) and C-ZnO (**d-f**); The frequency of 11-20 plane (**g**) and XRD patterns (**h**) of M-ZnO and C-ZnO.

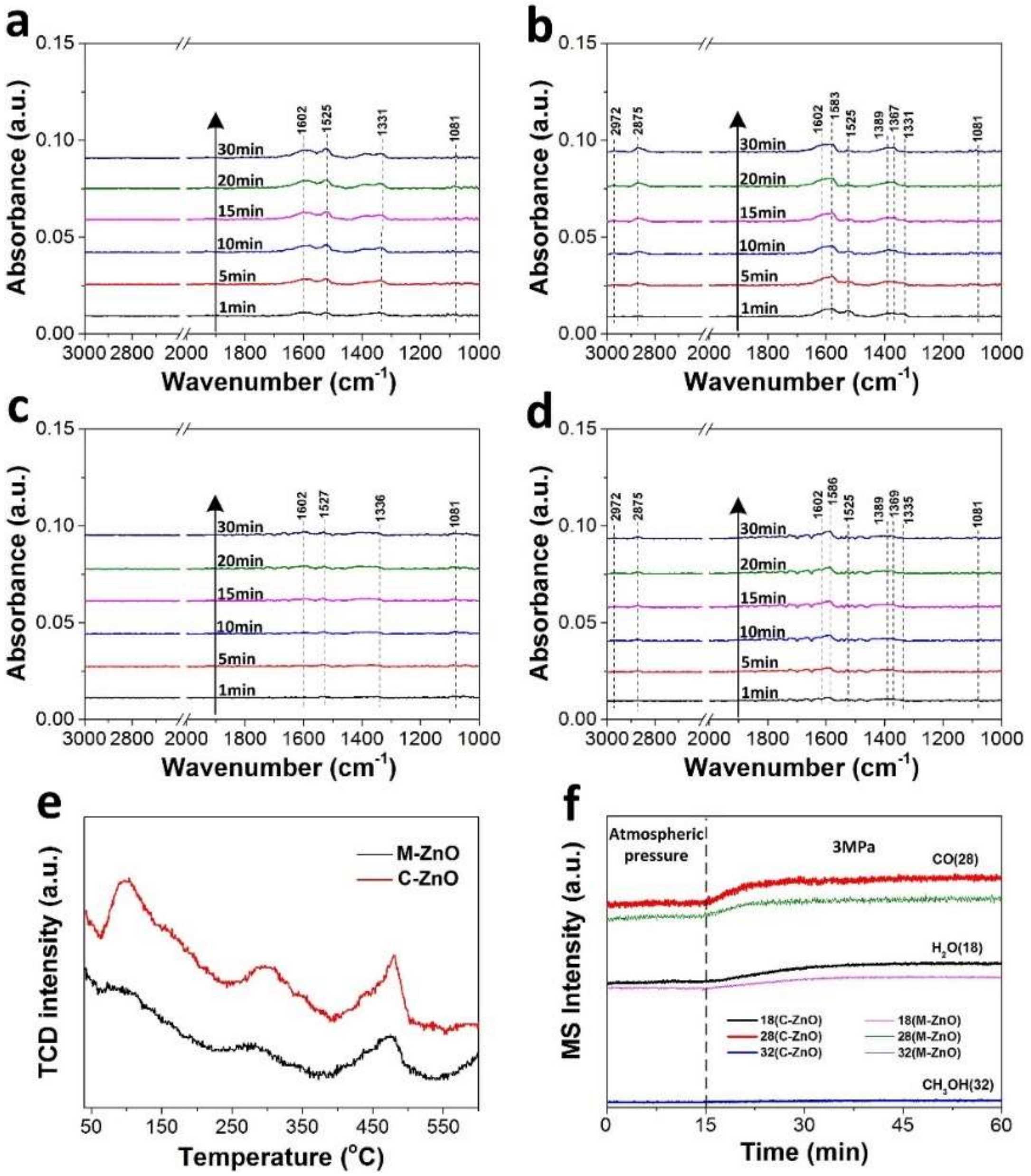

Supplementary Figure 20. *In situ* DRIFTS of C-ZnO and M-ZnO in CO_2 adsorption (**a** and **c**) and then switching to CO_2 + H_2 (**b** and **d**) at 220 °C under atmospheric pressure; CO_2-TPD profiles of C-ZnO and M-ZnO (**e**). MS analysis on the products of CO_2 + H_2 over C-ZnO and M-ZnO at 220 °C under different pressures (**f**).

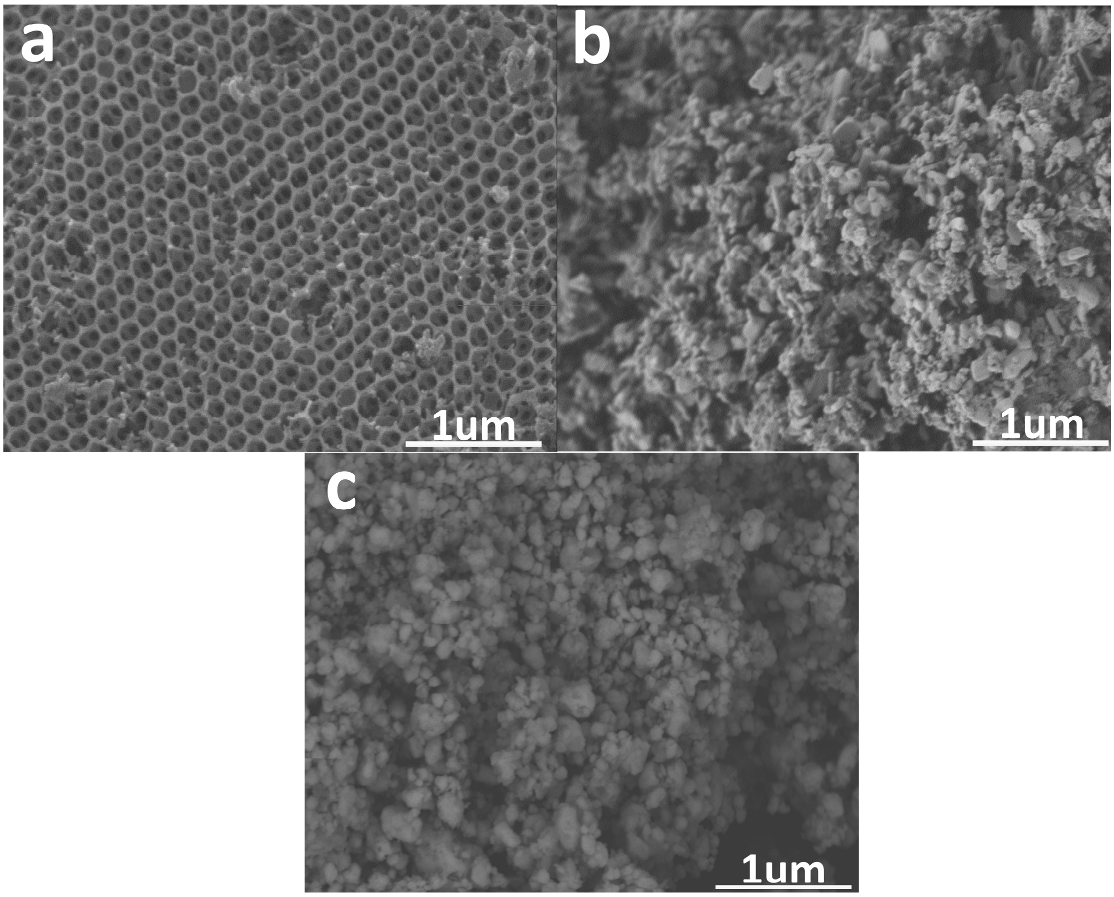

Supplementary Figure 21. SEM images of (a) M-CZZ(16), (b)N-CZZ(42) and (c) N-CZZ(16) samples.

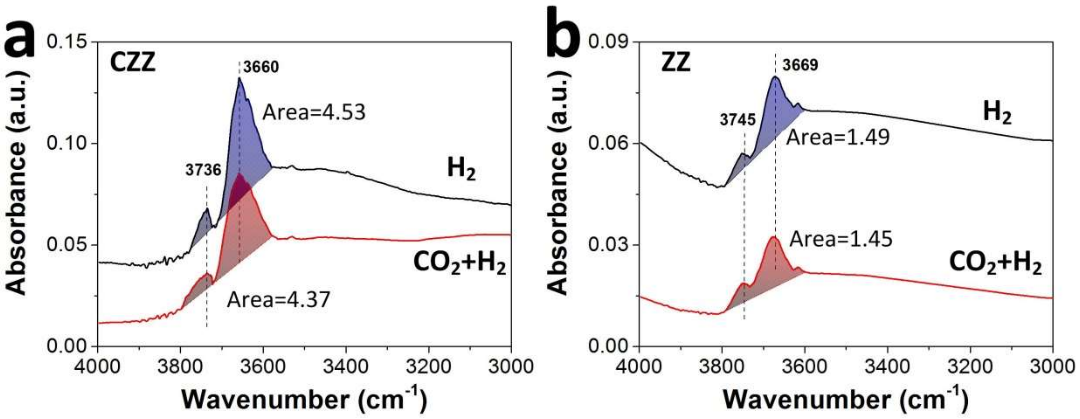

Supplementary Figure 22. *In-situ* DRIFTS of the hydroxyl group stretching region taken for CZZ (**a**) and ZZ (**b**) in H_2 and $CO_2 + H_2$, respectively. Spectra referenced to empty cell in He.

Additional *in situ* DRIFTS experiments were performed to determine the relatively degree of hydroxylation on the surface of the catalyst under reaction conditions. The hydroxyl groups on the surface of the CZZ and ZZ samples were measured in pure H_2 and CO_2+H_2 at 3MPa and 393K. Assuming that the surface of catalysts after pure H_2 reduction is likely covered with a saturation coverage of hydroxyl species, the ratio of the OH peak area in the CO_2+H_2 atmosphere to that in pure H_2 can be considered as a relatively degree of hydroxylation. As shown in Supplementary Figure 22, both samples show similar peak areas of hydroxyl in different atmospheres, suggesting that the catalysts show saturation coverage of OH (nearly 100% degree of hydroxylation) under the experiment conditions (3Mpa, 493 K and CO_2+3H_2). This supports the DFT model used in this manuscript.

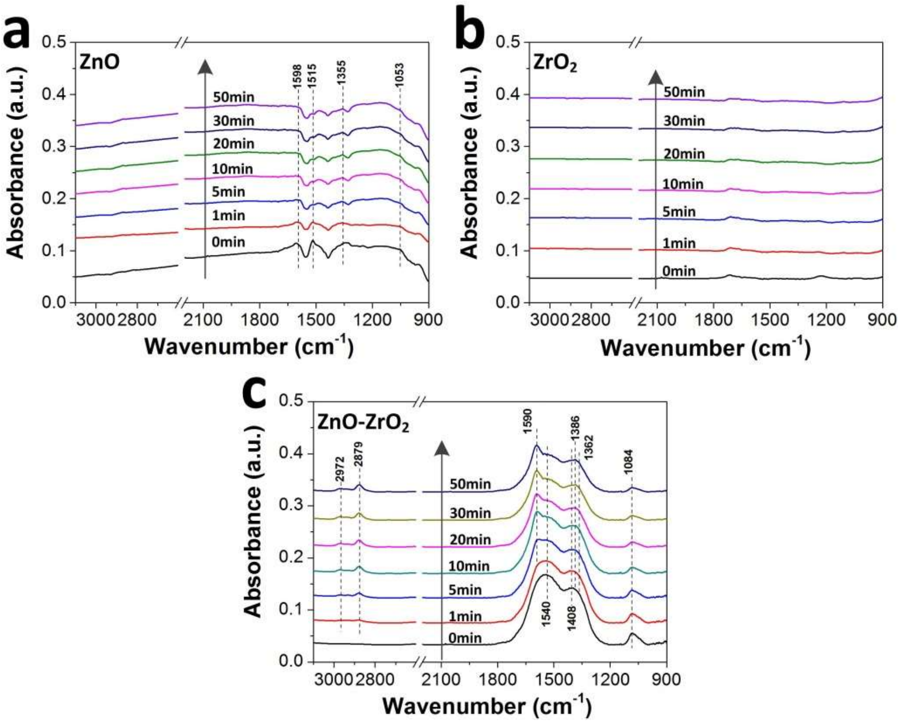

Supplementary Figure 23. *In situ* DRIFTS of ZnO (11) (a), ZrO_2(10) (b) and ZnO(15)-ZrO_2 (c) catalysts. Reaction conditions: CO_2+3H_2, 493 K and atmospheric pressure.

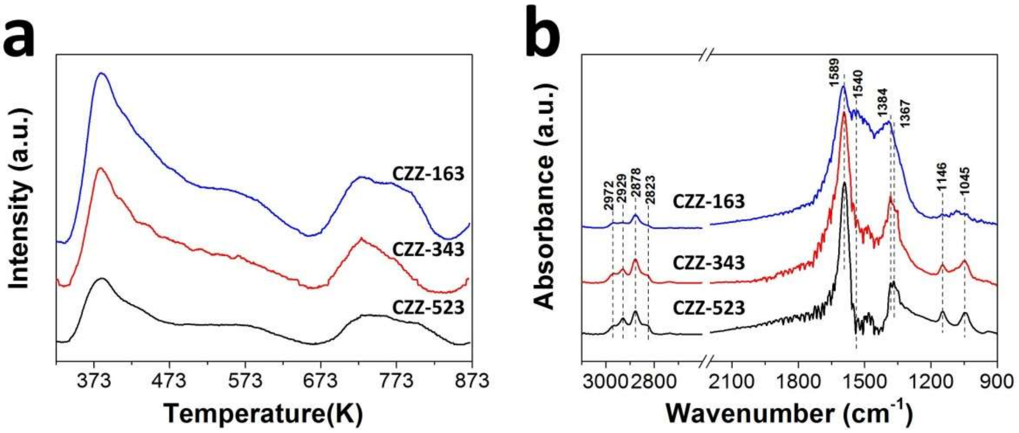

Supplementary Figure 24. CO_2-TPD profiles and *in situ* DRIFTS in CO_2+H_2 over CZZ catalysts with different compositions: CZZ-163, CZZ-343 and CZZ-523 means the Cu, ZnO, ZrO_2 molar ratio in the sample is 1:6:3, 3: 4:3, 5:2:3, respectively.

The comparison on the CO_2-TPD, *in situ* DRIFTS characterization, and catalytic activity over CZZ catalysts with different compositions was performed. Supplementary Figure 24 shows the CO_2-TPD profiles and the *in situ* DRIFTS, and the catalytic activity as well as the specific surface area/Cu specific surface area (S_{Cu}) are shown in Supplementary Table 6. It can be seen that the CO_2 desorption in CO_2-TPD (Supplementary Figure 24a) is enhanced and the bands of carbonate (1540 cm^{-1}) in the DRIFTS experiment also increases (Supplementary Figure 24b) with decreasing Cu content. This phenomenon supports the conclusion that the ZnO-ZrO_2 interface contributes to the CO_2 adsorption because the decrease of Cu content is accompanied by the increase of ZnO content which would result an increase in the ZnO-ZrO_2 interface “length”. On the other hand, the *in situ* DRIFTS experiments (Supplementary Figure 24b) show that the peak intensities of formate (2972, 2878, 1589, 1384, 1367 cm^{-1}) and methoxy (2929, 2823, 1146, 1045 cm^{-1}) decrease with the

decreasing Cu content. In addition, the formation of methanol is also suppressed by the decrease of Cu content (see Supplementary Table 6). This should be attributed to the fact that Cu related species are the active sites for H_2 dissociation, which provides active H for producing formate, methoxy and methanol. These phenomena further confirm the synergy among Cu, ZnO and ZrO_2 that the ZnO-ZrO_2 interface is the active sites of CO_2 adsorption and the Cu related species contribute to the dissociation of H_2. H_2 is dissociated on the Cu sites and then spilled to the ZnO-ZrO_2 interface to react with the carbon-containing intermediates to sequentially form formate, methoxy and methanol.

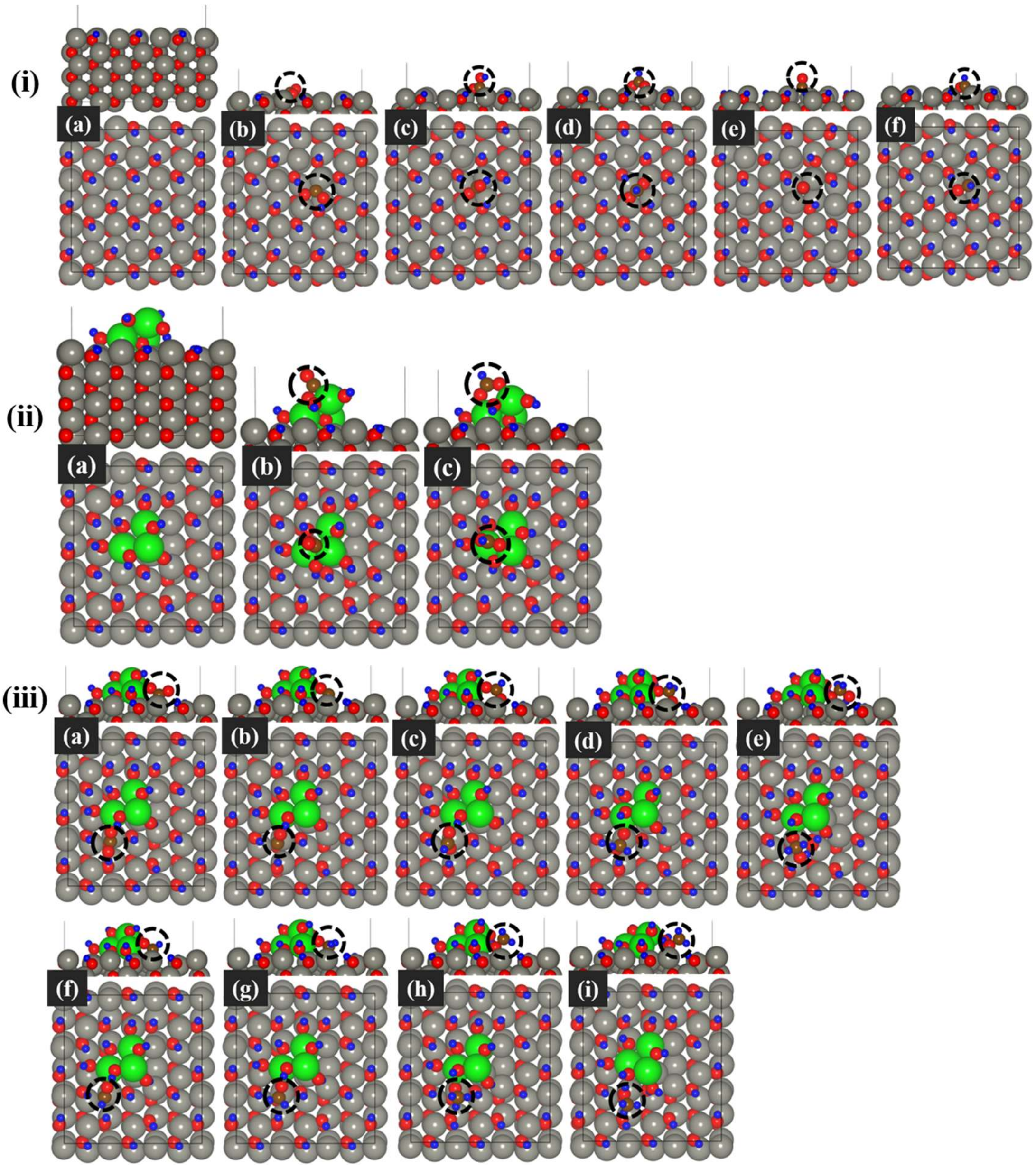

Supplementary Figure 25. DFT optimized structures. (i) ZnO(110): (a) ZnO(110) slab, (b) *CO_2, (c) *HOCO, (d) *HCOO, (e) *CO, and (f) *HCO. (ii) ZrO_2/ZnO(110): (a) ZrO_2 cluster on ZnO(110), (b) *CO_2 on ZrO_2 and (c) *HCOO on ZrO_2.(iii) adsorbates at ZrO_2/ZnO interface: (a) *CO_2, (b) *CO, (c) *HCOO, (d) *H_2COO, (e) *H_2COOH, (f) *HCO, (g) *H_2CO, (h) *H_3CO and (i) *CH_3OH. *(X) indicates adsorbed species. The reaction intermediates are shown inside the dotted circle. Gray: Zn, green: Zr, red: O, brown: C and blue: H

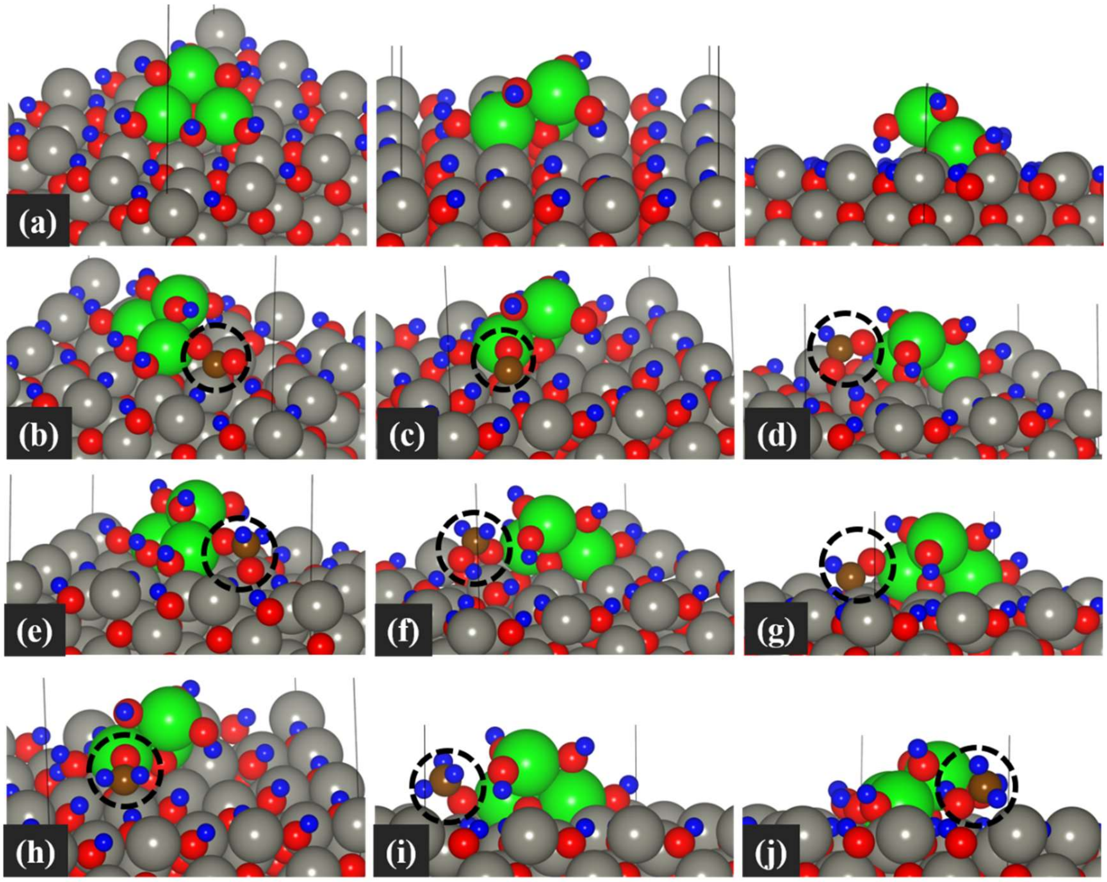

Supplementary Figure 26. DFT optimized structures. (a) ZrO_2/ZnO(110), (b) *CO_2, (c) *CO, (d) *HCOO, (e) *H_2COO, (f) *H_2COOH, (g) *HCO, (h) *H_2CO, (i) *H_3CO and (j) *CH_3OH. *(X) indicates adsorbed species. The reaction intermediates are shown inside the dotted circle. Gray: Zn, green: Zr, red: O, brown: C and blue: H

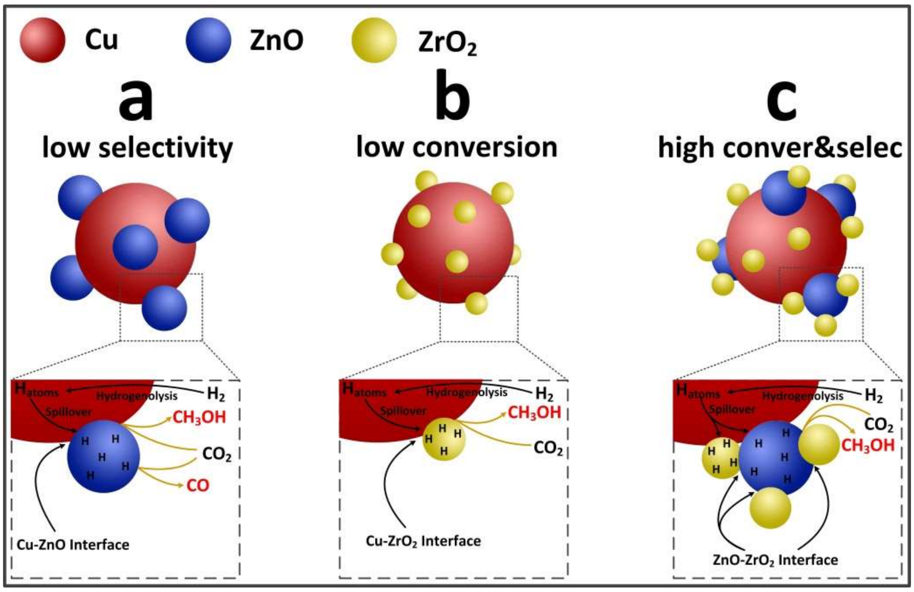

Supplementary Figure 27. Schematic diagram of the CO_2 hydrogenation process over (**a**) Cu-ZnO, (**b**) Cu-ZrO_2, and (**c**) Cu-ZnO-ZrO_2 catalysts

For the Cu-ZnO catalyst, the active sites of CO_2 hydrogenation to methanol are related to the Cu-ZnO species (Cu-ZnO interface or Cu-Zn alloy), while the hydrogenated ZnO is the active sites for CO_2 hydrogenation to CO[6]. The presence of abundant isolated ZnO results in relatively low methanol selectivity. For the Cu-ZrO_2 catalyst, the oxygen vacancies of ZrO_2 play a very important role for the conversion of CO_2 to methanol[7-9]. However, the oxygen vacancy concentration in ZrO_2, especially for tetragonal ZrO_2 (t-ZrO_2), is relatively low, which should be responsible for the low CO_2 conversion. In the case of the Cu-ZnO-ZrO_2 catalyst, the strong ZnO-ZrO_2 interaction creates more oxygen vacancies (as revealed by the XPS characterization shown in Supplementary Figure 17) that can enhance the CO_2 adsorption, contributing to the relatively high CO_2 conversion. In addition, the presence of ZrO_2 on ZnO reduces the surface proportion of exposed ZnO, which can eliminate the active sites for the reduction of CO_2 to CO, improving the methanol selectivity" .

Supplementary Table 1. Specific surface area (S_{BET}), Cu specific surface area (S_{Cu}), Cu grain size (d_{Cu}), Cu dispersion (D_{Cu}) and catalytic properties of the M-CZZ and N-CZZ samples.

Catalysts	S_{BET} (m^2/g)	d_{Cu} (nm)	S_{Cu} (m^2/g)[a]	D_{Cu} (%)	CO_2 conversion (%)[b]	MeOH selectivity (%)[b]	MeOH yield ($g_{MeOH}g_{Cata}^{-1}h^{-1}$)[b]
M-CZZ(16)	33.8	22.4	10.2	4.64	18.9	80.2	297.2
M-CZZ(19)	32.9	22.8	10.0	4.55	17.4	77.1	263.1
M-CZZ(24)	34.1	22.3	10.3	4.68	15.8	73.6	227.8
M-CZZ(36)	34.7	21.9	10.5	4.77	11.5	64.8	146.1
N-CZZ-(15)	36.7	22.4	10.3	4.68	14.2	59.3	160.3
N-CZZ-(25)	53.6	17.2	13.3	6.05	12.5	54.2	128.7
N-CZZ-(31)	45.2	23.6	9.7	4.41	11.3	51.4	110.5
N-CZZ-(43)	35.3	41.8	5.5	2.50	9.1	46.7	80.7

Reaction conditions: T=493 K, CO_2:H_2=1:3, P=3.0 MPa, WHSV=3 h^{-1}

Supplementary Table 2. Catalytic properties for TOF calculation and TOF values towards the M-CZZ and N-CZZ samples.

Catalysts	CO_2 conversion (%)	MeOH selectivity (%)	MeOH yield ($g_{MeOH}g_{Cata}^{-1}h^{-1}$)	TOF_{Cu} ($10^{-3}s^{-1}$)
M-CZZ(16)	9.7	91.4	582.2	20.6
M-CZZ(19)	8.9	89.7	517.8	18.1
M-CZZ(24)	7.8	84.5	426.6	15.7
M-CZZ(36)	5.8	73.6	279.4	9.9
N-CZZ-(15)	6.0	75.1	295.7	10.4
N-CZZ-(25)	5.6	65.3	237.9	6.5
N-CZZ-(31)	4.8	62.9	200.4	7.5
N-CZZ-(42)	3.3	56.2	145.8	9.7

Reaction conditions: T=493 K, CO_2:H_2=1:3, P=3.0 MPa, WHSV=10 h^{-1}

The TOF, which represents the molecular number of methanol formed per second per metallic copper atom, was calculated based on the S_{Cu} for all the catalysts. To ensure the reliability of TOF values and avoid the possible occurrence of thermodynamic constraints, the conversion should be controlled in low level (<10%). In this case, the WHSV is elevated to 10 h^{-1} for reducing CO_2 conversion.

Supplementary Table 3. Catalytic performance of some typical Cu-ZnO-ZrO_2 catalysts reported in literatures under similar conditions with that in the present work.

catalyst	H_2 : CO_2 ratio	Temperature (K)	Pressure (MPa)	Conversion (%)	Selectivity (%)	Velocity (ml/g/min)	Space-time yield (gMeOH/kgcata/h)
Cu-ZnO-ZrO_2[10]	3:1	513	3	17.0	41.5	40	48.8
Cu-ZnO-ZrO_2[11]	3:1	513	3	18.0	51.2	~166	302
Cu-ZnO-ZrO_2[12]	3:1	503	3	19.3	48.6	50	80
Cu-ZnO-ZrO_2[13]	3:1	513	3	11.8	46.0	~146	180
Cu-ZnO-ZrO_2[14]	3:1	513	3	17.5	48.4	~73	140
Cu-ZnO-ZrO_2[15]	3:1	523	3	19.4	29.3	~46	60
Cu-ZnO-ZrO_2[16]	3:1	503	5	15.4	66.8	~110	160
Cu-ZnO-ZrO_2[17]	3:1	493	5	7.9	64.0	160	162
Cu-ZnO-ZrO_2[18]	3:1	493	8	21.0	68.0	~55	181
Cu-ZnO-ZrO_2[19]	3:1	493	8	20.4	66.0	55	170
ZnO-ZrO_2[20]	3:1	593	5	10.0	86.0	400	730
This paper	3:1	493	3	18.2	80.2	100	297

Supplementary Table 4. XPS peaks fitting results of Zn Auger peak and O 1s over ZnO, ZrO_2, ZnO-ZrO_2.

Catalysts	The percentage of Zn Auger peak		Peak I/ Peak II ratio	The percentage of O 1s species		The ratio of OI/ OII
	Peak I (%)	Peak II (%)		OI (%)	OII (%)	
ZnO(15)-ZrO_2	26.7	73.3	0.36	30.7	69.3	0.44
ZnO(37)	14.3	85.7	0.16	20.8	79.2	0.26
ZnO(11)	16.4	83.6	0.19	22.1	77.9	0.28
ZrO_2(10)	-	-	-	20.3	79.7	0.25

Supplementary Table 5. Catalytic activity and surface area of Cu (S_{Cu}) for Cu-ZnO (molar ratio=5:2), Cu-ZrO_2 (molar ratio=5:3), Cu-ZnO-ZrO_2 (molar ratio=5:2:3) catalysts.

Catalysts	S_{BET} (m^2/g)	S_{Cu} (m^2/g)	CO_2 conversion (%)	MeOH selectivity (%)	MeOH yield (g/kg·h)	MeOH yield (mg/m^2·h)
Cu-ZnO	42.1	14.2	9.0	32.7	54.6	1.3
Cu-ZrO_2	36.8	8.6	3.8	58.7	46.2	1.2
Cu-ZnO-ZrO_2	53.6	13.3	12.5	54.2	128.7	2.3

Supplementary Table 6. The specific surface area (S_{BET}), the Cu specific surface area (S_{Cu}) and the catalytic activity of CZZ catalysts with different compositions: 163, 343 and 523 in the sample means the Cu, ZnO, ZrO_2 molar ratio is 1:6:3, 3: 4:3, 5:2:3, respectively.

Catalysts	S_{BET} (m^2/g)	S_{Cu} (m^2/g)	CO_2 conversion (%)	MeOH selectivity (%)	MeOH yield (g/kg·h)
CZZ-523	53.6	13.3	12.5	54.2	128.7
CZZ-343	60.9	8.2	10.9	49.6	101.2
CZZ-163	58.4	2.6	3.1	22.9	13.3

Supplementary Table 7. Comparison of catalytic activity for Cu-based catalysts with different Cu structures

Literatures	catalysts	S_{Cu} (m^2/g)	D_{Cu}(%)	TOF_{Cu} (methanol) ($10^{-3}s^{-1}$)
Guo et al. J. Catal. 2010, 271, 178.[21]	50-CZZ	3.32	-	11.8
	75-CZZ	1.20	-	15.0
	100-CZZ	0.75	-	17.8
	125-CZZ	1.26	-	14.6
	150-CZZ	1.50	-	14.4
Arena et al. J. Catal. 2007, 249, 185.[14]	Cu(12)/ZrO_2(6)	8.7	3.3	19.0
	Cu(11)ZnO(1)/ZrO_2(6)	17.4	6.2	11.7
	Cu(9)ZnO(3)/ZrO_2(6)	60.8	29.1	3.7
	Cu(3)ZnO(9)/ZrO_2(6)	44.7	57.9	3.9
Guo et al. J. Mol. Catal. A. 2011, 345, 60.[22]	CZ	2.83	-	5.0
	1%LCZ	3.22	-	4.7
	5%LCZ	4.36	-	4.5
	10%LCZ	3.55	-	5.4
Bonura et al. Appl. Catal. B. 2014, 152-153, 152.[11]	C6Z3Z1-CB	23	7.4	2.25
	C6Z3Z1-CT	18	6.3	2.20
	C6Z3Z1-OX	28	9.5	2.21
Słoczyński et al. Appl. Catal. A. 2003, 249,	CuZnZr	3.9	-	6.5
	CuZnZrMg	9.1	-	3.1

129.[23]	CuZnZrMn	13.5	-	2.6
Gao et al. J. Catal. 2013, 298, 51.[24]	CHTs-0	29.4	9.54	4.60
	CHTs-1	35.8	11.59	5.18
	CHTs-5	39.7	13.79	3.89
Karelovic et al. Catal. Sci. Technol. 2015, 5, 869.[25]	Cu(0.5)ZnO	0.40	12.3	0.95
	Cu(1)ZnO	0.67	9.8	0.87
	Cu(3)ZnO	0.75	4.2	0.83
	Cu(5)ZnO	0.86	2.8	0.84
	Cu(8)ZnO	0.25	0.5	4.05
	Cu(15)ZnO	0.32	0.4	3.77
Hu et al. J. Catal. 2018, 359, 17.[26]	CZ	7.2	-	2.53
	Pd-CZ-0.005	6.8	-	3.42
	Pd-CZ-0.01	5.0	-	11.8
	Pd-CZ-0.02	6.2	-	7.14
	Pd-CZ-0.03	2.7	-	13.5
	Pd-CZ-0.04	2.1	-	14.3
Valant et al. J. Catal. 2015, 324, 41.[3]	Zn/(Zn + Cu)=0.1	11	3.03	0.20
	Zn/(Zn + Cu)=0.3	9	3.03	0.93
	Zn/(Zn + Cu)=0.5	6	2.96	1.65
	Zn/(Zn + Cu)=0.62	5	3.03	2.77
	Zn/(Zn + Cu)=0.7	4	3.03	2.67
	Zn/(Zn + Cu)=0.9	1	3.10	3.81

Tisseraud et al. J. Catal. 2015, 330, 533.[6]	Zn/(Zn + Cu)=0.09	8	2.2	1.14
	Zn/(Zn + Cu)=0.3	10	3.4	1.50
	Zn/(Zn + Cu)=0.5	7	3.6	1.92
	Zn/(Zn + Cu)=0.7	13	10.6	0.89
	Zn/(Zn + Cu)=0.9	2	5.6	5.95

In the Cu-ZnO or Cu-ZrO_2 system, it is widely believed that bare Cu^0 metal is the origin of the reactivity for the hydrogenation of CO_2 to methanol, and a direct relationship between the Cu specific surface area (S_{Cu}) and the activity has been reported. However, many previous studies report that the oxide supports also play a very important role in the catalytic process. For Cu-ZnO catalysts, the oxygen vacancies in ZnO play a critical factor in determining the catalytic activity[3, 27]. The ZnO_x ($x < 1$) moieties are formed under reducing conditions at the Cu-ZnO interface, and this *in situ*-formed decorated surface is suggested to hold the active sites for methanol synthesis[28]. On the other hand, it has also been observed that the promoting effect of ZrO_2 on Cu/SiO_2 is due to the effective adsorption of CO_2 on ZrO_2[7, 29] . In addition, the results obtained by Koppel et al.[30] and Ma et al.[31] suggest that the presence of t-ZrO_2 on the Cu-ZrO_2 catalyst promotes the methanol formation from CO and CO_2. However, another view is that m-ZrO_2 is more active than t-ZrO_2 in the methanol formation[9, 32] due to the relatively high oxygen vacancy concentration on m-ZrO_2. Interestingly, Rhodes and Bell reported that when the surface area of Cu was higher than 2.5 m^2/g in the Cu-ZrO_2 catalysts, the further increase in the surface area of metallic Cu does not promote methanol synthesis and lowers the TOF value[21, 33]. In

summary, the catalytic activities depend not only on the S_{Cu} but also the physicochemical property of the supports.

It is more complicated for the ternary Cu-ZnO-ZrO_2 catalyst. As shown in Table S7, there are no obvious relationship between the Cu dispersion/Cu specific surface area and the TOF value. In the present work, Cu makes up the 3DOM framework of the microporous Cu-ZnO-ZrO_2 catalysts, and the microstructure of Cu particles (e.g., Cu dispersion, Cu specific surface area and Cu particle size) in all the samples is similar (see Supplementary Table 1). The only significant difference for the different samples is the particle size of ZnO. As shown in Figure 2a in the manuscript, both the TOF value and the selectivity of methanol decreases with increasing ZnO particle size. The ZnO particle size determines the Cu-ZnO interface "length", which further affects the dissociation of H_2 and the spillover efficiency of H. On the other hand, the ZnO particle size also influences the ZnO-ZrO_2 interface "length" that would modify the CO_2 adsorption capacity. Overall, both of the two types of interface "length" affect the catalytic activity.

Supplementary References

1. Kuld S, Conradsen C, Moses PG, Chorkendorff I, Sehested J. Quantification of Zinc Atoms in a Surface Alloy on Copper in an Industrial - Type Methanol Synthesis Catalyst. *Angew. Chem. Int. Et.* **53**, 5941 (2014).

2. Schott V*, et al.* Chemical activity of thin oxide layers: strong interactions with the support yield a new thin-film phase of ZnO. *Angew. Chem. Int. Et.* **52**, 11925-11929 (2013).

3. Valant AL, Comminges C, Tisseraud C, Canaff C, Pinard L, Pouilloux Y. The Cu–ZnO synergy in methanol synthesis from CO_2 , Part 1: Origin of active site explained by experimental studies and a sphere contact quantification model on Cu + ZnO mechanical mixtures. *J. Catal.* **324**, 41-49 (2015).

4. Lunkenbein T, Schumann J, Behrens M, Willinger MG. Formation of a ZnO Overlayer in Industrial Cu/ZnO/Al_2O_3 Catalysts Induced by Strong Metal-Support Interaction. *Angew. Chem. Int. Et.* **54**, 4544-4548 (2015).

5. Sudarsanam P, Mallesham B, Reddy PS, Großmann D, Grünert W, Reddy BM. Nano-Au/CeO_2 catalysts for CO oxidation: Influence of dopants (Fe, La and Zr) on the physicochemical properties and catalytic activity. *Appl. Catal. B* **144**, 900-908 (2014).

6. Tisseraud C*, et al.* The Cu–ZnO synergy in methanol synthesis from CO_2 , Part 2: Origin of the methanol and CO selectivities explained by experimental studies and a sphere contact quantification model in randomly packed binary mixtures on Cu–ZnO coprecipitate catalysts. *J. Catal.* **330**, 533-544 (2015).

7. Fisher IA, Bell AT. In-Situ Infrared Study of Methanol Synthesis from H_2 /CO_2 over Cu/SiO_2 and Cu/ZrO_2/SiO_2. *Journal of Catalysis* **172**, 222-237 (1997).

8. Pokrovski K, Jung KT, Bell AT. Investigation of CO and CO_2 adsorption on tetragonal and monoclinic zirconia. *Langmuir* **17**, 4297-4303 (2001).

9. Rhodes MD, Bell AT. The effects of zirconia morphology on methanol synthesis from CO and H_2 over Cu/ZrO_2 catalysts : Part I. Steady-state studies. *J. Catal.* **233**, 198-209 (2005).

10. Xiao J, Mao D, Guo X, Yu J. Effect of TiO_2, ZrO_2 and TiO_2-ZrO_2 on the performance of CuO-ZnO catalyst for CO_2 hydrogenation to methanol. *Appl. Surf. Sci.* **338**, 146-153 (2015).

11. Bonura G, Cordaro M, Cannilla C, Arena F, Frusteri F. The changing nature of the active site of Cu-Zn-Zr catalysts for the CO_2 hydrogenation reaction to methanol. *Appl. Catal. B* **152-153**, 152-161 (2014).

12. Li C, Yuan X, Fujimoto K. Development of highly stable catalyst for methanol synthesis from

carbon dioxide. *Appl. Catal. A* **469**, 306-311 (2014).

13. Bonura G, Arena F, Mezzatesta G, Cannilla C, Spadaro L, Frusteri F. Role of the ceria promoter and carrier on the functionality of Cu-based catalysts in the CO_2 to methanol hydrogenation reaction. *Catal. Today* **171**, 251-256 (2011).

14. Arena F, Barbera K, Italiano G, Bonura G, Spadaro L, Frusteri F. Synthesis, characterization and activity pattern of Cu–ZnO/ZrO_2 catalysts in the hydrogenation of carbon dioxide to methanol. *J. Catal.* **249**, 185-194 (2007).

15. Raudaskoski R, Niemelä MV, Keiski RL. The effect of ageing time on co-precipitated Cu/ZnO/ZrO_2 catalysts used in methanol synthesis from CO_2 and H_2. *Topics in Catal.* **45**, 57-60 (2007).

16. Dong X, Li F, Zhao N, Xiao F, Wang J, Tan Y. CO_2 hydrogenation to methanol over Cu/ZnO/ZrO_2 catalysts prepared by precipitation-reduction method. *Appl. Catal. B* **191**, 8-17 (2016).

17. Arena F, Mezzatesta G, Zafarana G, Trunfio G, Frusteri F, Spadaro L. Effects of oxide carriers on surface functionality and process performance of the Cu–ZnO system in the synthesis of methanol via CO_2 hydrogenation. *J. Catal.* **300**, 141-151 (2013).

18. Słoczyński J*, et al.* Catalytic activity of the M/(3ZnO·ZrO_2) system (M=Cu, Ag, Au) in the hydrogenation of CO_2 to methanol. *Appl. Catal. A* **278**, 11-23 (2004).

19. Słoczyński J*, et al.* Effect of metal oxide additives on the activity and stability of Cu/ZnO/ZrO_2 catalysts in the synthesis of methanol from CO_2 and H_2. *Appl. Catal. A* **310**, 127-137 (2006).

20. Wang J*, et al.* A highly selective and stable ZnO-ZrO_2 solid solution catalyst for CO_2 hydrogenation to methanol. *Sci. Adv.* **3**, e1701290 (2017).

21. Guo X, Mao D, Lu G, Wang S, Wu G. Glycine–nitrate combustion synthesis of CuO-ZnO-ZrO_2 catalysts for methanol synthesis from CO_2 hydrogenation. *J. Catal.* **271**, 178-185 (2010).

22. Guo X, Mao D, Lu G, Wang S, Wu G. The influence of La doping on the catalytic behavior of Cu/ZrO_2 for methanol synthesis from CO_2 hydrogenation. *J. Mol. Catal. A* **345**, 60-68 (2011).

23. Słoczyński J*, et al.* Effect of Mg and Mn oxide additions on structural and adsorptive properties of Cu/ZnO/ZrO2 catalysts for the methanol synthesis from CO_2. *Appl. Catal. A* **249**, 129-138 (2003).

24. Gao P*, et al.* Influence of Zr on the performance of Cu/Zn/Al/Zr catalysts via hydrotalcite-like

precursors for CO_2 hydrogenation to methanol. *J. Catal.* **298**, 51-60 (2013).

25. Karelovic A, Ruiz P. The role of copper particle size in low pressure methanol synthesis via CO2 hydrogenation over Cu/ZnO catalysts. *Catal. Sci. Tech.* **5**, 869-881 (2015).

26. Hu B, Yin Y, Liu G, Chen S, Hong X, Tsang SCE. Hydrogen spillover enabled active Cu sites for methanol synthesis from CO_2 hydrogenation over Pd doped CuZn catalysts. *J. Catal.* **359**, 17-26 (2018).

27. Grunwaldt JD, Molenbroek AM, Topsøe NY, Topsøe H, Clausen BS. In Situ Investigations of Structural Changes in Cu/ZnO Catalysts. *J. Catal.* **194**, 452-460 (2000).

28. Vesborg PCK*, et al.* Transient behavior of Cu/ZnO-based methanol synthesis catalysts. *J. Catal.* **262**, 65-72 (2009).

29. Schilke TC, Fisher IA, Bell AT. In Situ Infrared Study of Methanol Synthesis from CO_2/H_2 on Titania and Zirconia Promoted Cu/SiO_2. *J. Catal.* **184**, 144-156 (1999).

30. Köppel RA, Stöcker C, Baiker A. Copper- and Silver–Zirconia Aerogels: Preparation, Structural Properties and Catalytic Behavior in Methanol Synthesis from Carbon Dioxide. *J. Catal.* **179**, 515-527 (1998).

31. Ma ZY, Yang C, Wei W, Li WH, Sun YH. Catalytic performance of copper supported on zirconia polymorphs for CO hydrogenation. *J. Mol. Catal. A* **231**, 75-81 (2005).

32. Jung KT, Bell AT. Effects of Zirconia Phase on the Synthesis of Methanol over Zirconia-Supported Copper. *Catal. Let.* **80**, 63-68 (2002).

33. Rhodes MD, Pokrovski KA, Bell AT. The effects of zirconia morphology on methanol synthesis from CO and H_2 over Cu/ZrO_2 catalysts: Part II. Transient-response infrared studies. *J. Catal.* **233**, 210-220 (2005).
