# Supplementary material for: Exploring the ternary interactions in Cu–ZnO–ZrO2 catalysts for efficient CO2 hydrogenation to methanol
Source: Nat Commun. 2019 Mar 11;10:1166. doi: 10.1038/s41467-019-09072-6 (PMC6411953; doi:10.1038/s41467-019-09072-6)
Supplement: Supplementary file 2 — Peer Review File [file 41467_2019_9072_MOESM2_ESM.pdf]

Editorial Note: This manuscript has been previously reviewed at another journal that is not operating a transparent peer review scheme. This document only contains reviewer comments and rebuttal letters for versions considered at *Nature Communications*.

Reviewers' comments:

Reviewer #1 (Remarks to the Author):

The article deals with a very "hot" topic - valorization of CO2 into methanol. The catalysts of this reaction are being constantly improved, the mechanism is under investigation, many works are connected to the possible implementation and its industrial potential.

Even if the most active catalysts are known, the mechanism and the action of catalyst's parts is under questions. The authors claim the mechanistic study of the ternary effect between catalyst's components (Cu, Zn and Zr oxides). The authors insist in the title on the synergy between the 3 oxides, they also insists on the synergy in CO2 adsorption, they say that the synegry effect is the key of CO2 conversion and MeOH selectivity. To explane the nature of those synergetic actions this only postulate is used: "The synergy among Cu, ZnO and ZrO2 can be ascribed to that the ZnO-ZrO2 interface is responsible for the adsorption and activation of CO2, and the Cu related species contribute to the dissociative adsorption of hydrogen..."

In my opinion this postulate says about two different actions, no real prove of "synergy" or "ternary effect" that the authors insist so much in the title and in the body of tha article.

Despite the general quality of the results on quite high level I am affraid that the article in this shape is imprecise and not siutable for publication.

Reviewer #2 (Remarks to the Author):

The manuscript described the hydrogenation of CO2 over 3DOM Cu-Zn-ZrO2 catalyst and found that the catalyst exhibited the best catalytic performance, 18% conversion of CO2 and the 80% selectivity to metahnol. The reaction mechanism was investigated by using in-situ techniques at the reaction conditions. I read the revised manuscript and the corresponding letter and found that the author has revised the manuscript carefully according to the reviewers' commends. The result is interesting and I would like to recommend it to be published in this journal after minor revision.

(1) In situ IR result showed that the carbonate species were converted into the formate species, no CO intermediate. At this time, the selectivity to methanol is about 80%, not 100%. why?

(2) In Fig3, The carbonate species decrease quickly over Cu-ZnO, Cu-ZrO2, no formate species was observed. Is the carbonate species converted into CO which was desorbed quickly?

Exploring the ternary interactions in Cu-ZnO-ZrO_2 catalysts for efficient CO_2 hydrogenation to methanol

Yuhao, Wang[1,2†], Shyam Kattel[3†], Wengui Gao[1,2], Kongzhai Li[1,4*], Ping Liu[3], Jingguang G. Chen[3, 5*], Hua Wang [1*]

▪ Response to Reviewers

We thank the reviewers for their thoughtful comments, which are valuable in improving the quality of our manuscript. As described below, we have made a detailed explanation and performed additional experiments to address all the comments.

▪ Reviewer #1

The article deals with a very "hot" topic - valorization of CO_2 into methanol. The catalysts of this reaction are being constantly improved, the mechanism is under investigation, many works are connected to the possible implementation and its industrial potential.

Even if the most active catalysts are known, the mechanism and the action of catalyst's parts is under questions. The authors claim the mechanistic study of the ternary effect between catalyst's components (Cu, Zn and Zr oxides). The authors insist in the title on the synergy between the 3 oxides, they also insists on the synergy in CO_2 adsorption, they say that the synergy effect is the key of CO_2 conversion and MeOH selectivity. To explain the nature of those synergetic actions this only postulate is used: "The synergy among Cu, ZnO and ZrO_2 can be ascribed to that the ZnO-ZrO_2 interface is responsible for the adsorption and activation of CO_2, and the Cu related species contribute to the dissociative adsorption of hydrogen..."

In my opinion this postulate says about two different actions, no real prove of "synergy" or "ternary effect" that the authors insist so much in the title and in the body of tha article.

Despite the general quality of the results on quite high level I am afraid that the article in this shape is imprecise and not suitable for publication.

[Response]

The conclusion on the ternary synergistic action among Cu, ZnO and ZrO_2 are

based on the combined results of catalytic activity, *in-situ* DRIFTS experiments and DFT calculations. As shown in Figure R1a, no methanol is detected over the $ZnO-ZrO_2$ catalyst for CO_2 hydrogenation, but both Cu-ZnO and $Cu-ZrO_2$ catalysts show good activity for this reaction. This indicates that the Cu/ZnO and Cu/ZrO_2 interactions are crucial for the methanol synthesis. On the other hand, the $Cu-ZnO-ZrO_2$ ternary catalyst exhibits much higher methanol yield than either Cu-ZnO or $Cu-ZrO_2$ even though it shows lower surface area of Cu (S_{Cu}) than the Cu/ZnO catalyst (see Figure R1b), suggesting that the $ZnO-ZrO_2$ interaction should also play an important role in the $Cu-ZnO-ZrO_2$ catalyst for CO_2 hydrogenation. The higher activity of the $Cu-ZnO-ZrO_2$ catalyst is a clear indication of the synergistic action among Cu, ZnO and ZrO_2.

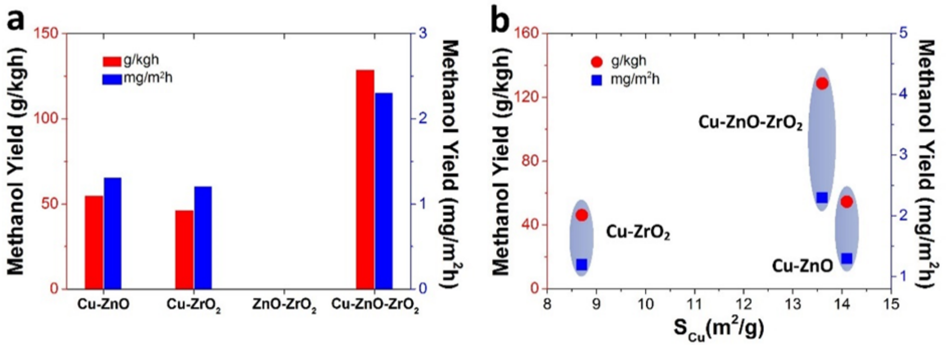

Figure R1. (**a**) Methanol yields (normalized by specific surface area or normalized by weight) of Cu-ZnO, $Cu-ZrO_2$, $ZnO-ZrO_2$ and $Cu-ZnO-ZrO_2$ catalysts for CO_2 hydrogenation and (**b**) the relationship between S_{Cu} and methanol yield.

The further evidence for the ternary synergy is observed by the *in-situ* DRIFTS experiments. Figures R2 compares the evolutions of IR spectra over Cu-ZnO, $Cu-ZrO_2$, $ZnO-ZrO_2$ and $Cu-ZnO-ZrO_2$ catalysts after switching feed gas from CO_2 to H_2 in the reaction cell under atmosphere pressure. Carbonate species (the peaks in range from ~1550 to ~1050 cm^{-1} for the black IR spectra) can be detected over all four catalysts in the presence of CO_2, but they quickly disappear after switching feed gas from CO_2 to H_2 for the Cu-ZnO (Figures R2a) and $Cu-ZrO_2$ (Figures R2b) samples and no obvious new species are observed. On the other hand, it is very interesting that the carbonate species on $ZnO-ZrO_2$ sample are gradually weakened during this process, which is accompanied by the formation of formate species (~2972, 2878, 1593, 1386, 1362 cm^{-1}). More importantly, for the $Cu-ZnO-ZrO_2$ catalysts (Figure R2d), the transformation of carbonate species to formate species is also detected, but they are furhter converted to methoxy speceis (2930, 2821, 1147, 1046 cm^{-1}) in the prsence of H_2 (which is not dected on $ZnO-ZrO_2$). These phenomena

indicate that the $ZnO-ZrO_2$ may bind the formate intermediates and the presence of Cu could promote the further hydrogenation of formate speceis to methoxy speceis, which generally accepted as the last intermediate for methanol generation from CO or CO_2 hydrogenation *[refs: Behrens et al. Science 2012, 336, 893; Graciani et al. Science 2014, 345, 546; Kattel et al. Science 2017, 357, 1296; Kuld et al. Science 2016, 352, 969.]*.

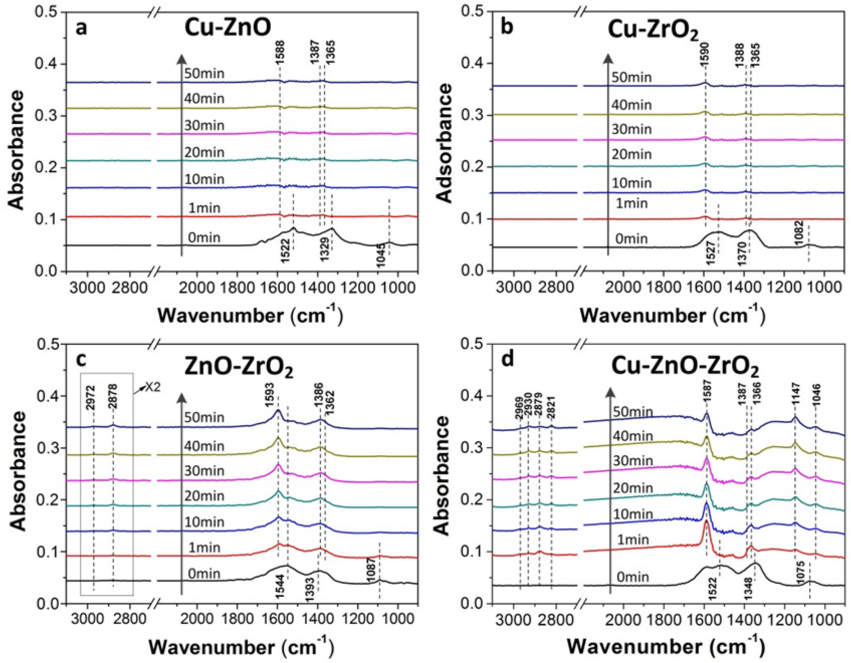

Figure R2. Comparison on the surface species on (**a**) Cu-ZnO, (**b**) $Cu-ZrO_2$, (**c**) $ZnO-ZrO_2$ and (**d**) $Cu-ZnO-ZrO_2$ systems in the designed conditions. *In-situ* DRIFT spectra over different catalysts at 493 K after switching feed gas from CO_2 (after introducing CO_2 into the reaction camber for 10 min) to H_2 with a flow rate of 40 mL/min under atmosphere pressure.

The DFT calculations also support the FRIFTS findings. As shown in Figure 5A of the manuscript, the reaction intermediates prefer the ZrO_2-ZnO interface rather than either oxide alone. For instance, the CO_2 adsorption adopts a conformation with C bound with Zn and one of O anchored on Zr. Such configuration provides a stronger binding energy (-2.32 eV) than that on ZnO-Cu(111) (-0.13 eV), ZrO_2-Cu(111) (-1.18 eV), ZrO_2 cluster on ZnO(110) (-1.95 eV) and ZnO(110) (-1.94 eV), indicating that the ZrO_2/ZnO interface facilitates the activation and transformation of CO_2, a key step for CO_2 activation.

Although the Cu component was not specifically considered in the DFT calculations, the H_2 dissociative at the $ZnO-ZrO_2$ interface is an endothermic process (ΔE = 0.47 eV), which is less favorable than that at the Cu-oxide interface (ΔE = -0.33 eV). It suggests that the presence of Cu is necessary to allow the facile

formation of *H at the Cu-oxide interface under reaction conditions, which facilitates the subsequent hydrogenation processes by providing the surface *H species.

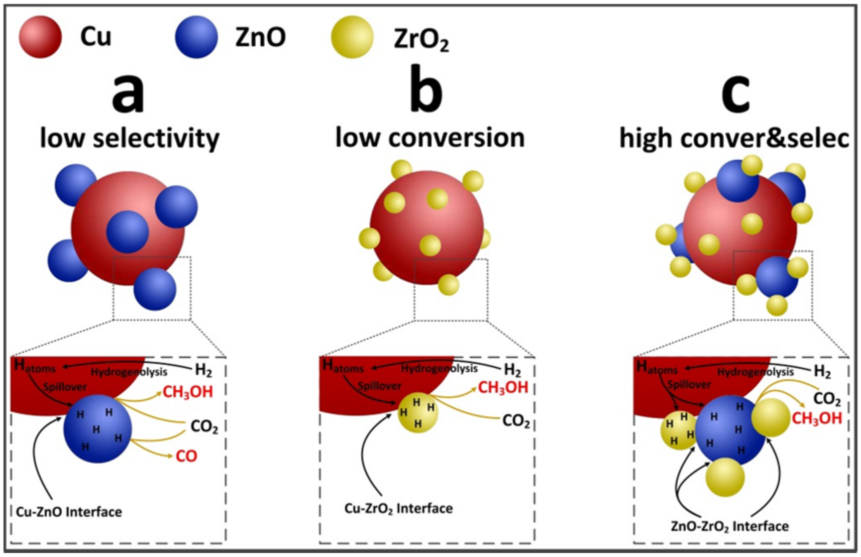

Figure R3. Schematic diagram of the CO_2 hydrogenation process over (**a**) Cu-ZnO, (**b**) Cu-ZrO_2, and (**c**) Cu-ZnO-ZrO_2 catalysts

Figure R3 shows a schematic diagram for the differences in catalytic activity (the detail data of the catalytic activity can be found in Tables S1, S2 and S5) and reaction pathways among Cu-ZnO, Cu-ZrO_2 and Cu-ZnO-ZrO_2 catalysts for CO_2 hydrogenation to methanol. The reaction pathway for Cu-ZnO and Cu-ZrO_2 catalysts are based on the findings in related references. For the Cu-ZnO catalyst, the active sites of CO_2 hydrogenation to methanol are related to the Cu-ZnO species (Cu-ZnO interface or Cu-Zn alloy), while the hydrogenated ZnO is the active sites for CO_2 hydrogenation to CO *[Tisseraud et al. J. Catal. 2015, 330, 533]*. The presence of abundant isolated ZnO results in relatively low methanol selectivity. For the Cu-ZrO_2 catalyst, the oxygen vacancies of ZrO_2 could improve the Cu-ZrO_2 interaction and the CO_2 adsorption ability play a very important role for the conversion of CO_2 to methanol *[Fisher et al. J. Catal., 1997, 172, 222; Pokrovski et al. Langmuir, 2001, 17, 4297; [Rhodes et al. J. Catal., 2005, 233, 198]*. However, the oxygen vacancy concentration in ZrO_2, especially for tetragonal ZrO_2 (t-ZrO_2), is relatively low, which should be responsible for the low CO_2 conversion. In the case of the Cu-ZnO-ZrO_2 catalyst, the strong ZnO-ZrO_2 interaction creates more oxygen vacancies (as revealed by the XPS characterization shown in Figure S17 in the Supplementary Information) that can enhance the CO_2 adsorption, contributing to the relatively high CO_2 conversion. In addition, the presence of ZrO_2 on ZnO reduces the surface proportion of exposed ZnO, which eliminates the active sites for the reduction of CO_2 to CO, improving the

methanol selectivity.

Overall, the Cu-ZnO-ZrO_2 catalyst shows much higher activity for CO_2 hydrogenation to methanol than the binary catalyst systems, and the interplay among ZnO, ZrO_2 and Cu is essential to enable the high conversion of CO_2 and high selectivity toward methanol. The ZnO-ZrO_2 interaction contributes to the adsorption of CO_2 and binds the formate intermediate, and the interaction of Cu with the ZnO-ZrO_2 support provides the hydrogen source for the further step hydrogenations of intermediate species to methanol.

[Action]

Even though our results clearly show that the ternary Cu-ZnO-ZrO_2 catalyst demonstrates enhanced rate for methanol production over the corresponding binary catalysts, we understand the caution raised by the Reviewer. Based on the Reviewer's suggestion we have decided to put less emphasis on the term "ternary synergy". Accordingly, we have changed "ternary synergistic action" to "ternary interactions" in the title of the manuscript; we have also changed "ternary synergy" to "ternary interactions" in the Abstract and Introduction. Such changes would allow the readers to reach unbiased conclusions regarding the origin of the unique catalytic properties of the Cu-ZnO-ZrO_2 catalyst.

We have also rewritten the final discussion to emphasize on the strong interplay among Cu, ZnO and ZrO_2 in pages 21 and 22:

"For the Cu/ZnO system, the Cu-ZnO interface or the Cu-Zn surface alloy is considered as the active sites for CO_2 hydrogenation to methanol[3,5-7,24,28,29]. In the case of Cu/ZrO_2, the Cu-ZrO_2 interface plays a very important role for methanol formation[10, 26, 27, 33]. For both the binary catalysts, the catalytic activity is determined by the Cu-ZnO or Cu-ZrO_2 interaction that is closely related to the physicochemical features (e.g., Cu particle size and surface area of Cu) of Cu spices. As shown in the comparison of the catalytic activity of Cu/ZnO, Cu-ZrO_2 and Cu-ZnO-ZrO_2 in Figure S13, the Cu-ZnO-ZrO_2 ternary catalyst exhibits much higher methanol yield than either Cu-ZnO or Cu-ZrO_2 even though it shows a lower surface area of Cu (S_{Cu}) than the Cu/ZnO catalyst, suggesting that the ZnO-ZrO_2 interaction should also play an important role in the Cu-ZnO-ZrO_2 catalyst for CO_2 hydrogenation. Combining the results of XPS (Figure S17) and CO_2-TPD (Figure 4d), it can be concluded that the ZnO-ZrO_2 interaction promotes the formation of oxygen vacancies, which should be the active sites for CO_2 adsorption. The *in-situ* DRIFTS (Figures 3 and S12) experiments reveal that the ZnO-ZrO_2 interface is crucial for the transformation of

carbonate to formate during CO_2 hydrogenation. However, no surface methoxy, which is a crucial intermediate species for methanol synthesis, is detected on the ZnO-ZrO_2 catalyst (see Figures 4a-c), while it is abundant on the Cu-ZnO-ZrO_2 catalysts (see Figures S5 and S9). These results indicate that the presence of Cu is necessary for the formate hydrogenation to methoxy in methanol synthesis from CO_2+H_2. It is reasonable to propose that, in the Cu-ZnO-ZrO_2 system, the ZnO-ZrO_2 interaction contributes to the adsorption of CO_2 and binds the formate intermediate, and the interaction of Cu with the ZnO-ZrO_2 support is responsible for the dissociative adsorption of hydrogen and the subsequent hydrogenation of carbonaceous intermediate species (e.g., formate and methoxy) to methanol." Figure S27 shows an illustration to emphasize on the role of Cu, ZnO and ZrO_2 in the ternary interaction and a full discussion is also provided.

In addition, Figures R1and R3 were added into the Supplementary Information as Figure S13 and S27, respectively. The related discussions were also added into the SI as follows:

"As shown in Figure S13a, no methanol is detected over the ZnO-ZrO_2 catalyst for CO_2 hydrogenation, but both Cu-ZnO and Cu-ZrO_2 catalysts show good activity for this reaction. This indicates that the Cu/ZnO and Cu/ZrO_2 interactions are crucial for the methanol synthesis. On the other hand, the Cu-ZnO-ZrO_2 ternary catalyst exhibits much higher methanol yield than either Cu-ZnO or Cu-ZrO_2 even though it shows a lower surface area of Cu (S_{Cu}) than the Cu/ZnO catalyst (see Figure S13b), suggesting that the ZnO-ZrO_2 interaction should also play an important role in the Cu-ZnO-ZrO_2 catalyst for CO_2 hydrogenation."

"For the Cu-ZnO catalyst, the active sites of CO_2 hydrogenation to methanol are related to the Cu-ZnO species (Cu-ZnO interface or Cu-Zn alloy), while the hydrogenated ZnO is the active sites for CO_2 hydrogenation to CO[17]. The presence of abundant isolated ZnO results in relatively low methanol selectivity. For the Cu-ZrO_2 catalyst, the oxygen vacancies of ZrO_2 play a very important role for the conversion of CO_2 to methanol[18-20]. However, the oxygen vacancy concentration in ZrO_2, especially for tetragonal ZrO_2 (t-ZrO_2), is relatively low, which should be responsible for the low CO_2 conversion. In the case of the Cu-ZnO-ZrO_2 catalyst, the strong ZnO-ZrO_2 interaction creates more oxygen vacancies (as revealed by the XPS characterization shown in Figure S17) that can enhance the CO_2 adsorption, contributing to the relatively high CO_2 conversion. In addition, the presence of ZrO_2 on ZnO reduces the surface proportion of exposed ZnO, which can eliminate the active sites for the reduction of CO_2 to CO, improving the methanol selectivity"

- **Reviewer #2**

The manuscript described the hydrogenation of CO_2 over 3DOM Cu-Zn-ZrO_2 catalyst and found that the catalyst exhibited the best catalytic performance, 18% conversion of CO_2 and the 80% selectivity to methanol. The reaction mechanism was investigated by using in-situ techniques at the reaction conditions. I read the revised manuscript and the corresponding letter and found that the author has revised the manuscript carefully according to the reviewers' commends. The result is interesting and I would like to recommend it to be published in this journal after minor revision.

Comment 1: In situ IR result showed that the carbonate species were converted into the formate species, no CO intermediate. At this time, the selectivity to methanol is about 80%, not 100%. why?

[Response to Comment 1]

The related phenomenon can be found in Figures 3 and S5, which is performed by switching the CO_2 feed gas (after introducing CO_2 into the reaction camber for 10 min) to H_2 at 493 K and 0.1 MPa. Under this condition, the formed carbonate species were converted into the formate species without the presence of CO. However, the 80% selectivity of methanol is obtained in the activity testing of catalysts that is performed in the flow of CO_2/H_2 mixture at 3.0 MPa. Under this condition, the apparent gaseous CO (2175 and 2115 cm^{-1}) and methanol are observed in the *in-situ* DRIFTS experiment as shown in Figure 2b, which is consistent with the catalytic activity testing. This indicates that the reaction pressure is crucial for the kinetics of CO_2 hydrogenation. The reaction products (CO and methanol) of CO_2 hydrogenation are hardly detected by IR at low reaction pressure (0.1 MPa).

[Action]

We added the following sentence on page 10 to emphasize the effect of pressure on methanol production:

"It is also noted that no CO intermediate is detected during the DRIFTS experiment under atmospheric pressure (see Figure S5), which is inconsistent with the formation of CO in the CO_2 hydrogenation at 3.0 MPa (see Figure 2). This reveals that the reaction pressure also afftects the production of CO from CO_2."

Comment 2: In Fig3, the carbonate species decrease quickly over Cu-ZnO, Cu-ZrO_2, no formate species was observed. Is the carbonate species converted into CO which was desorbed quickly?

[Response to Comment 2]

According to the literatures *[Graciani et al. Science 2014, 345, 546; Kattel et al. Science, 2017, 355, 1296; Kattel et al. J. Am. Chem. Soc. 2016, 138, 12440; Yang et al. J. Phys. Chem. C 2012, 116, 248; Yang et al. J. Phys. Chem. C 2010, 114, 17205; Yang et al. J. Catal. 2013, 298, 10; Grabow et al. ACS Catal. 2011, 1, 365.]*, the CO intermediate in CO_2 hydrogeneration is originated from carboxylate (*COOH) decomposition. In the present work, Figures 3a and S11a reveal that it is difficult to detect the intermediate on the Cu-ZnO sample under 493K, probably due to fast reaction. To reduce the reaction rare, the *in-situ* DRIFTS excrements at a lower temperature (273K) was performed, as shown in Figure R4. The bands at 1630, 1420 and 1207 cm^{-1} are assigned to $\nu_{as}(CO_3)$, $\nu_s(CO_3)$ and δ(OH) of bidentate bicarbonate species*[Chen et al. ACS Catal. 2015, 5, 1653; Köck et al. J. Phys. Chem. C 2013, 117, 17666]*, respectively, and the bands at 1529, 1322 and 1057 cm^{-1} related to $\nu_{as}(CO_3)$ and $\nu_s(CO_3)$ of bidentate carbonate species are also detected. The $\nu_{as}(CO_3)$ and $\nu_s(CO_3)$ of monodentate carbonate species are observed at 1484 and 1365 cm^{-1}, respectively. It is very noteworthy that the apparent band at 1225 cm^{-1} is detected at 1 min and then disappears at 5 min, concurrent with the onset of a band at 2014 cm^{-1} that is attributed to the adsorbed CO. In the *in-situ* DRIFTS measurements, no C-H vibration (3000-2800 cm^{-1}) is detected at 1min, indicating the absence of formate species. Therefore, the band at 1225 cm^{-1} should be attribute to carboxylate species, which is unstable and can be converted into CO. In this case, it is possible that the carbonate species is rapidly converted to carboxylate species and then to CO, as suggested by the Reviewer.

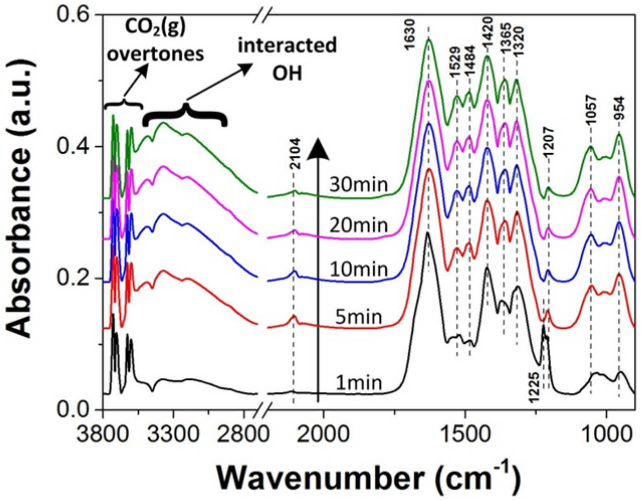

Figure R4. *In-situ* DRIFT spectra after switching the feed from He to 25%CO_2/H_2 over Cu-ZnO catalyst. Reaction condition: 273 K, atmospheric pressure, 40 mL/min.

REVIEWERS' COMMENTS:

Reviewer #1 (Remarks to the Author):

The article combine the experimental and theoretical studies on the Cu-Zn-Zr catalyst for CO2 hydrogenation to methanol which is on of the hot topics in the area of CO2 valorisation. Binary combinations of Cu-Zn, Zn-Zr and Cu-Zr are helpful to understand the interactions in the ternary catalyst. The findings proposed in this work enrich the understanding of the fundamental features of the CO2 hydrogenation catalysts and could be helpful for designing complex catalysts with multiple active components.
Regarding the corrections made and the detailed comments provided by the authors the manuscript could be acceptable for publication.

Reviewer #2 (Remarks to the Author):

The author has revised the manuscript carefully according to the reviewers' commends and suggestions, I would like to recommend it to be published in nthis journal at the present form.
